# Inferring sex-specific demographic history from SNP data

**DOI:** 10.1371/journal.pgen.1007191

**Published:** 2018-01-31

**Authors:** Florian Clemente, Mathieu Gautier, Renaud Vitalis

**Affiliations:** 1 Institut de Biologie Computationnelle (IBC), Univ. Montpellier, CNRS, Montpellier, France; 2 CBGP, INRA, CIRAD, IRD, Montpellier SupAgro, Univ. Montpellier, Montpellier, France; University of California Berkeley, UNITED STATES

## Abstract

The relative female and male contributions to demography are of great importance to better understand the history and dynamics of populations. While earlier studies relied on uniparental markers to investigate sex-specific questions, the increasing amount of sequence data now enables us to take advantage of tens to hundreds of thousands of independent loci from autosomes and the X chromosome. Here, we develop a novel method to estimate effective sex ratios or ESR (defined as the female proportion of the effective population) from allele count data for each branch of a rooted tree topology that summarizes the history of the populations of interest. Our method relies on Kimura’s time-dependent diffusion approximation for genetic drift, and is based on a hierarchical Bayesian model to integrate over the allele frequencies along the branches. We show via simulations that parameters are inferred robustly, even under scenarios that violate some of the model assumptions. Analyzing bovine SNP data, we infer a strongly female-biased ESR in both dairy and beef cattle, as expected from the underlying breeding scheme. Conversely, we observe a strongly male-biased ESR in early domestication times, consistent with an easier taming and management of cows, and/or introgression from wild auroch males, that would both cause a relative increase in male effective population size. In humans, analyzing a subsample of non-African populations, we find a male-biased ESR in Oceanians that may reflect complex marriage patterns in Aboriginal Australians. Because our approach relies on allele count data, it may be applied on a wide range of species.

## Author summary

The history of populations and their social organization is often intricate due to breeding structures, migration patterns or population bottlenecks. Estimation of the female proportion of the effective population (sex ratio) is therefore important to better understand this underlying social structure and dynamics. This question has been mainly investigated so far by comparing genetic variation of mitochondrial DNA and the Y chromosome, two uniparentally inherited markers that reflect the demographic history of females and males, respectively. To overcome the intrinsic limitations of these genetic markers, and to take advantage of the increasing amount of sequence data, we propose a new approach that uses large numbers of independent polymorphisms from autosomes and the X chromosome to estimate sex ratios, throughout the history of populations. This method allows us to confirm a strongly female-biased sex ratio in modern dairy and beef cattle breeds. Yet, we find a strongly male-biased sex ratio during domestication times, consistent with an easier taming and management of cows, and/or introgression from wild auroch males. Analyzing human data from a sample of non-African populations, we find a male bias in Oceanians, possibly indicating complex marriage patterns among Aboriginal Australian groups.

## Introduction

In dioecious species, contrasting patterns of genetic differentiation between males and females provide important information on social organization [1], dispersal and mating patterns [2, 3], and demographic history [4]. Some correlation may exist between the adult sex ratio and behavior [5]: in bird species with female-biased adult sex ratio, for instance, males have multiple mates and females care for their offspring, while the opposite has been observed in species with male-biased sex ratio [6]. The proportion of females can also provide information about the reproductive potential of a population, which is essential for wildlife management of endangered species [7].

To date, the characterization of sex-specific genetic variation has mainly been based on uniparentally inherited markers: mitochondrial DNA (mtDNA), which is transmitted by females to their offspring, and the non-recombining portion of the Y chromosome (NRY), which is inherited through the male line only [8–14]. However, due to the lack of recombination in both mtDNA and NRY, the potential influence of other evolutionary forces, in particular selection, challenge the interpretation of the observed patterns of genetic diversity [15–17]. To circumvent this problem, an alternative approach has been proposed, which consists in comparing the amount of genetic variation at both autosomal and X-linked markers [18]. Because they recombine, autosomes and X chromosomes harbor markers that may only be locally affected by selection. Such markers are therefore highly informative about demographic differences between males and females [15], as was shown from the inference of sex-specific processes from the analysis of microsatellite markers [1, 3, 18], single nucleotide polymorphisms (SNPs) [19–21] and sequence data [22, 23].

In an isolated, random mating population with constant size and separate sexes, the effective population size for X-linked genes is expected to be three-quarters of that for autosomal genes, when the numbers of females and males are equal [24–26]. If the numbers of females and males are not equal, however, the ratio of X-to-autosome effective size is expected to deviate from three-quarters. This suggests that an effective sex ratio (ESR), defined as the female proportion of the effective population, can be inferred from the X-to-autosome ratio of genetic diversity [24, 27]. Accordingly, Hammer et al. [22] estimated the ratio of X-to-autosome effective size from observed levels of diversity, and found an excess of X-linked diversity in six geographically diverse human populations. They interpreted their findings as reflecting the widespread effect of larger female than male effective population sizes in humans. Labuda et al. [23] proposed to estimate the female-to-male breeding ratio from patterns of linkage disequilibrium (LD) on the X chromosome and the autosomes in humans. Although the original approach was undermined by errors in their mathematical derivations [28, 29], a reanalysis based on corrected equations [28] supported Hammer et al.’s [22] claim of an excess of breeding females in human history. This LD-based method is not affected by the choice of DNA segments as entire chromosomes are considered. However, the method is only applicable to species for which detailed and reliable linkage maps are available. In yet another approach, Keinan et al. [19] derived an estimator of the ratio of X-to-autosome effective size across pairs of populations, based on measures of differentiation (*F*_ST_). Contrary to Hammer et al. [22], they found indirect evidence of a male-biased ESR in the lineage ancestral to the split between European and Asian populations, coinciding with the Out-of-Africa expansion. This apparent paradox [26] was reconciled by Emery et al. [30], who showed that *F*_ST_-based approaches are more sensitive to recent events, whereas approaches measuring nucleotide diversity likely respond to older signals in the data. Finally, all aforementioned methods infer contemporary, population-specific ESR and hence provide only indirect information about historical ESR. Altogether, these arguments point to the difficulty of estimating past changes in the ESR.

Here, we present a hierarchical Bayesian model to estimate contemporary and ancestral ESR in multiple populations, and therefore, to identify historical changes in sex-specific demography. More precisely, the demographic history of populations is represented as a multifurcating tree, and the ESR is inferred for each branch of that tree. Our approach makes full use of the information contained in genome-wide SNP data and can be applied to a wide range of model and non-model species, i.e. it does not require a detailed and reliable linkage map. Instead of relying on summary statistics (as in [19, 21, 22, 30]), we explicitly model the change in allele frequencies along each branch of the population tree, using Kimura’s time-dependent diffusion approximation [31]. Our method is an extension to the model by Gautier and Vitalis [32], taking advantage of the joint analysis of autosomal and X-linked allele frequency data.

The motivation behind our study is threefold: (i) to improve the original model to yield unbiased estimates of the branch lengths, particularly for internal branches; (ii) to extend the model and provide estimates of branch lengths for both autosomal and X-linked data, and therefore to infer the ESR; and (iii) to evaluate our method through simulations and provide real data application examples from cattle and human. In the following, we show that parameters are inferred robustly even under scenarios that violate some of the model assumptions. In cattle, as expected from the breeding scheme, our method detects a strongly female-biased ESR in both dairy and beef commercial cattle breeds, and a moderately female-biased ESR in African cattle. Conversely, we observed a strongly male-biased ESR during early domestication times, consistent with an easier taming and management of cows, and/or introgression from wild auroch males, that would both cause a relative increase in male effective population size. In humans, the analysis of a subset of whole-genome sequence data recently published by Pagani et al. [33], provides evidence for a male-biased ESR in Oceanian human populations, that may result from complex marriage patterns among Aboriginal Australian groups.

## Results

### Model

The starting point for our model is detailed in Gautier and Vitalis [32], and implemented in the software package KimTree. In short, KimTree is a hierarchical Bayesian model, where the allele frequencies are modeled along each branch of a population tree that needs to be specified a priori, using Kimura’s time-dependent diffusion approximation for genetic drift [31].

Consider a sample of *I* populations sharing a common history, represented as a tree. Each population has a label, *i*, which varies from 1 to *I* for the sampled populations, and from *I* + 1 to *r* for the internal nodes of the tree, where *r* represents the population at the root of the tree (i.e., the most ancestral population in the tree). In the following, we denote *a*(*i*) as the ancestral population of population *i*. The data consist of *J* bi-allelic SNPs. Let *n*_*ij*_ be the total number of genes sampled at the *j*th locus in the *i*th population. Let *y*_*ij*_ be the corresponding observed count of the reference allele, which is arbitrarily defined. Assuming Hardy-Weinberg Equilibrium (HWE), the conditional distribution $P(y_{ij}|n_{ij},x_{ij})$ of *y*_*ij*_ given *n*_*ij*_ and the (unknown) allele frequency *x*_*ij*_ is binomial. In the absence of mutation, assuming that population *i* with effective size *N*_e,*i*_ diverged from *a*(*i*) for *t*_*i*_ discrete non-overlapping generations, the distribution *π*_*K*_(*x*_*ij*_ ∣ *x*_*a*(*i*)*j*_, *τ*_*i*_) of *x*_*ij*_, conditional upon the allele frequency *x*_*a*(*i*)*j*_ in the parental population, and upon the branch length *τ*_*i*_ ≡ *t*_*i*_/(2*N*_e,*i*_) is given by Kimura’s time-dependent diffusion approximation (see Eqs 4.9 and 4.16 in Kimura [31]). In Gautier and Vitalis [32], the prior distribution *π*(*x*_*rj*_) of the frequency *x*_*rj*_ of the reference allele for the *j*th SNP in the root population follows a beta distribution Beta(1.0, 1.0), and the branch lengths *τ*_*i*_’s are assumed to be sampled from a uniform distribution with support from 10^−4^ to 10. Assuming that genetic drift occurs independently in each branch of the tree (i.e., there is no migration between branches), we may characterize the gene frequency hierarchically along the tree from the most ancestral population toward the leaves. The full model then takes the form:

$$\begin{array}{ccc} \pi (X,\tau |Y,N) & \propto & \left[\prod_{i=1}^{I}\prod_{j=1}^{J}P(y_{ij}|n_{ij},x_{ij})\right]\\ & \times & [\prod_{i=1}^{r-1}\pi (\tau_{i})\prod_{j=1}^{J}\pi_{K}(x_{ij}|x_{a(i)j},\tau_{i})]\prod_{j=1}^{J}\pi (x_{rj}), \end{array}$$ (1)

where **X** ≡ (*x*_*ij*_) is a matrix of allele frequencies for all populations and loci, **Y** ≡ (*y*_*ij*_) is a matrix of observed allele counts for all sampled populations and loci, **N** ≡ (*n*_*ij*_) is the corresponding matrix of total allele counts, and ***τ*** ≡ (*τ*_*i*_) is a vector of branch lengths. In the present study, the model has been improved in several directions. First, we extended KimTree to estimate the hyper-parameters of the Beta(*α*, *β*) prior for allele frequencies in the root population. Estimating the hyper-parameters of the beta distribution allows for a more flexible allele frequency distribution at the root, potentially shifting the total age of the tree. Following Gautier [34], we re-parameterized the beta distribution using hyper parameters *μ* ≡ *α*/(*α* + *β*) and *ν* ≡ (*α* + *β*). We assumed a uniform prior for *μ* with support from 0 to 1 and an exponential prior for *ν*, i.e. μ∼U(0,1) and *ν* ∼ exp(1.0), respectively.

Second, we extended the model to account for the fact that the dataset consists, by construction, of polymorphic sites only. In SNP datasets, indeed, sites that are fixed across the entire sample have been filtered out. This is a non-trivial issue, since the fraction of sites that are monomorphic in the sample, but were polymorphic in the root population, contains information on the branch lengths. Ignoring this information may therefore result in biased estimates of the branch lengths. A solution to this problem is to condition the likelihood on SNP polymorphism, which is achieved by defining an indicator variable λ_*j*_, which equals 1 if the *j*th position is polymorphic in the full sample (0 < ∑_*i*_ *y*_*ij*_ < ∑_*i*_ *n*_*ij*_). Using this formalism, we can then compute the probability for a given SNP to be polymorphic across the sampled populations, conditionally on the topology, the branch lengths, and the allele frequencies in the root population. Here, we use a coalescent argument to compute this probability, as detailed in the Materials and methods section.

Last, the model was extended to jointly analyze allele frequencies from both autosomal and X-linked markers. In a single, isolated population (here, along each branch in the tree), the effective size for autosomal markers and X-linked markers (here expressed as numbers of diploid individuals) may be computed from the relative genetic contribution of both sexes (males and females) to the future of the population: $N_{e}^{\left(A\right)}=4N_{e}^{f}N_{e}^{m}/(N_{e}^{f}+N_{e}^{m})$, and $N_{e}^{\left(X\right)}=9N_{e}^{f}N_{e}^{m}/(2N_{e}^{f}+4N_{e}^{m})$ (see Eq 8.10 and 8.12 in Wright [24]). Defining the ESR as: $\xi \equiv N_{e}^{f}/(N_{e}^{f}+N_{e}^{m})$, these equations can be recast as: $N_{e}^{\left(A\right)}=4\xi (1-\xi )(N_{e}^{f}+N_{e}^{m})$ and $N_{e}^{\left(X\right)}=9\xi (1-\xi )(N_{e}^{f}+N_{e}^{m})/(4-2\xi )$. Since the branch lengths are measured on a diffusion time scale, they must be defined independently for each genetic system (X or A), and therefore read: $\tau^{\left(A\right)}\equiv t/\left(2N_{e}^{\left(A\right)}\right)$ and $\tau^{\left(X\right)}\equiv t/\left(2N_{e}^{\left(X\right)}\right)$. Rearranging the above expressions, it follows that the ESR can be written as:

$$\begin{array}{c} \xi =2-\frac{9}{8}\frac{\tau^{\left(X\right)}}{\tau^{\left(A\right)}} \end{array}$$ (2)

**Fig 1:**
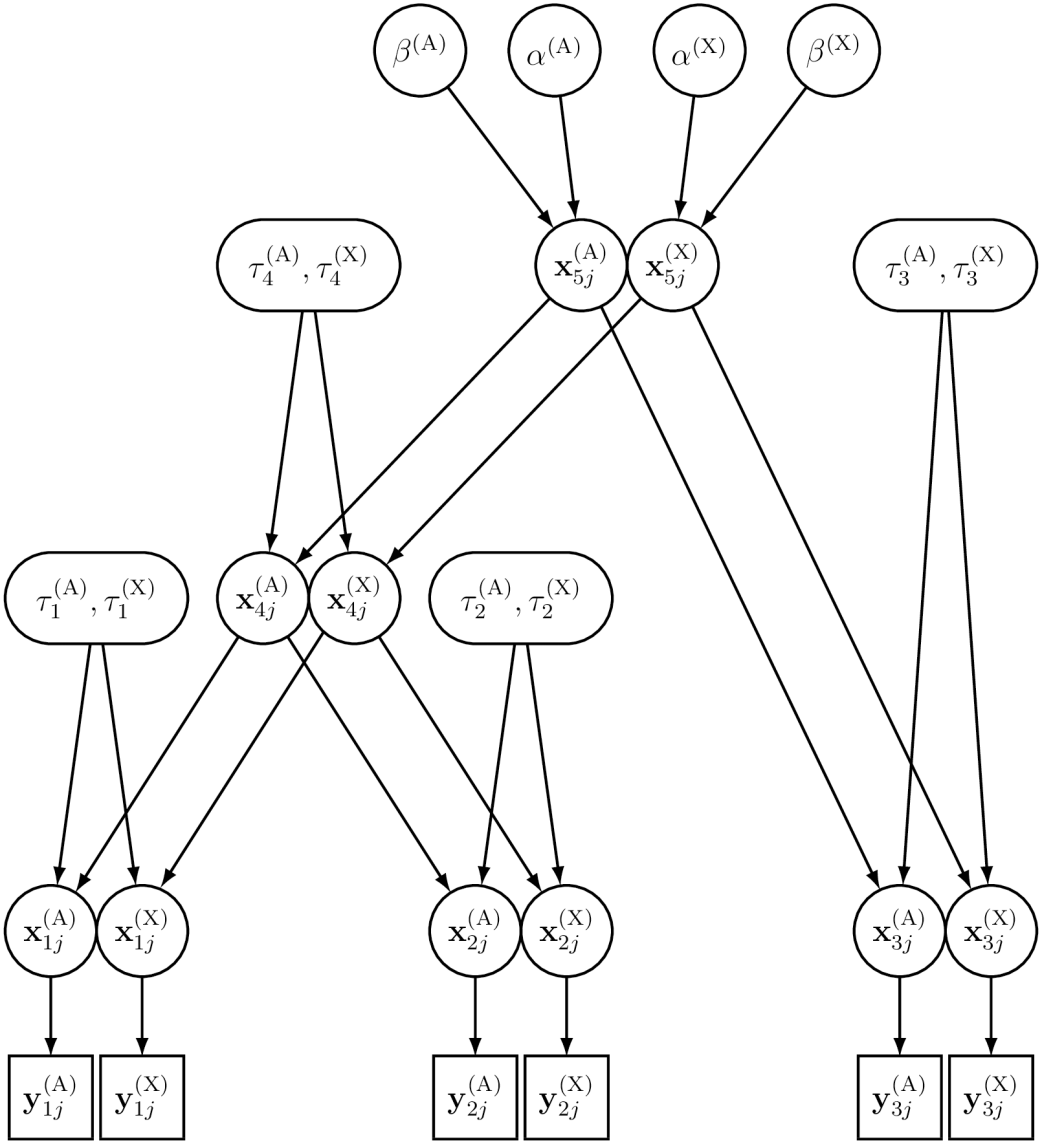
Directed acyclic graph (DAG) of the hierarchical Bayesian model for a three-population example tree. The square nodes characterize the data, i.e. $y_{ij}^{\left(\Omega \right)}\left(\Omega \in \left\{A,X\right\}\right)$ represents the observed allele counts from autosomal and X-linked data in population *i* at SNP *j*. The circles and rounded rectangles represent the parameters to be estimated: $x_{ij}^{\left(\Omega \right)}$ is the (unknown) allele frequency in population *i*; $\tau_{i}^{\left(\Omega \right)}\equiv t/(2N_{i}^{\left(\Omega \right)})$ is the length (in a diffusion time scale) of the branch leading to population *i*; *α*^(Ω)^ and *β*^(Ω)^ are the shape and scale parameters of the beta distribution, which describes the allele frequency distribution in the root population. Unidirectional edges (arrows) represent direct stochastic relationships within the model. They indicate the conditional dependency between connected nodes.

In principle, it would be possible to analyze the data from both genetic systems independently, and compute the ESR in each branch of the tree from the posterior distributions of the branch lengths for autosomal and X-linked markers. However, this would ignore the constraints that tie the effective sizes (and hence the branch lengths) of both genetic systems, since 0 < *ξ* < 1 (see S1 Fig). Therefore, we defined a new model that allows to borrow information from the prior constraints (see Fig 1), where all the parameters are specific to one or the other genetic system. In the following, we use the index Ω for the genetic system (Ω ∈ {A, X}).

In this new model, as in Eq (1), the reference allele counts $y_{ij}^{\left(\Omega \right)}$ follow a binomial distribution $P(y_{ij}^{\left(\Omega \right)}|n_{ij}^{\left(\Omega \right)};x_{ij}^{\left(\Omega \right)})$, given the (unknown) allele frequencies $x_{ij}^{\left(\Omega \right)}$ at the leaf nodes and the total number $n_{ij}^{\left(\Omega \right)}$ of genes sampled at the *j*th locus (*j* = 1, …, *J*^(Ω)^) in the *i*th population. The reference allele frequency for any given SNP *j* along the branches of the tree is assumed to follow Kimura’s time-dependent diffusion approximation $\pi_{K}\left(x_{ij}^{\left(\Omega \right)}|x_{a(i)j}^{\left(\Omega \right)},\tau_{i}^{\left(\Omega \right)}\right)$, conditional upon the ancestral reference allele frequency *x*_*a*(*i*)*j*_ in the parental population and upon the branch length $\tau_{i}^{\left(\Omega \right)}\equiv t_{i}/\left(2N_{e,i}^{\left(\Omega \right)}\right)$ (see Eqs 4.9 and 4.16 in Kimura [31]). At the highest hierarchical level of the model (see S1 Fig), the reference allele frequency at the root node is assumed to follow a beta distribution $\pi (x_{rj}^{\left(\Omega \right)}|\alpha^{\left(\Omega \right)},\beta^{\left(\Omega \right)})$ with hyper-parameters *α*^(Ω)^ and *β*^(Ω)^. The full joint posterior distribution of the model parameters Θ ≡ {**X**^(A)^,**X**^(X)^, ***τ***^(A)^, ***τ***^(X)^, *α*^(A)^, *α*^(X)^, *β*^(A)^, *β*^(X)^}, given the data $D\equiv \{Y^{\left(A\right)},Y^{\left(X\right)},N^{\left(A\right)},N^{\left(X\right)}\}$, therefore reads:

$$\begin{array}{ll} \pi \left(\Theta ,\lambda =1|D\right) & \propto [\prod_{\Omega \in \{\text{A},\text{X}\}}\left(\prod_{i=1}^{I}\prod_{j=1}^{J^{\left(\Omega \right)}}P(y_{ij}^{\left(\Omega \right)}|n_{ij}^{\left(\Omega \right)},x_{ij}^{\left(\Omega \right)})\right)\\ & \times \left(\prod_{i=1}^{r-1}\prod_{j=1}^{J^{\left(\Omega \right)}}\pi_{K}(x_{ij}^{\left(\Omega \right)}|x_{a(i)j}^{\left(\Omega \right)},\tau_{i}^{\left(\Omega \right)})\right)\\ & \times \left(\prod_{j=1}^{J^{\left(\Omega \right)}}\pi (x_{rj}^{\left(\Omega \right)}|\alpha^{\left(\Omega \right)},\beta^{\left(\Omega \right)})\right)\pi (\alpha^{\left(\Omega \right)})\pi (\beta^{\left(\Omega \right)})]\left(\prod_{i=1}^{r-1}\pi (\tau_{i}^{\left(\text{A}\right)},\tau_{i}^{\left(\text{X}\right)})\right)\\ & \times {\left(\prod_{j=1}^{J^{\left(\Omega \right)}}\;P(\lambda_{j}^{\left(\Omega \right)}=1|\alpha^{\left(\Omega \right)},\beta^{\left(\Omega \right)},\tau^{\left(\Omega \right)},n_{j}^{\left(\Omega \right)})\right)}^{-1} \end{array}$$ (3)

Since all markers are polymorphic, by definition, we assume that $\lambda \equiv \{\lambda_{j}^{\left(A\right)},\lambda_{j}^{\left(X\right)}\}=1$ (unit vector of length *J*^(A)^ + *J*^(X)^). This model follows from Eq (1), except that the square brackets integrate over the two genetic systems. One can also note that the parameters of the beta distribution of allele frequencies at the root node are estimated (see the first terms in the third line of the above equation). Furthermore, the prior distribution of the branch lengths lies outside the square brackets, since $\pi (\tau_{i}^{\left(A\right)},\tau_{i}^{\left(X\right)})$ represents the joint prior distribution for the branch lengths (see the Materials and methods section). Last, $P(\lambda_{j}^{\left(\Omega \right)}=1|\alpha^{\left(\Omega \right)},\beta^{\left(\Omega \right)},\tau^{\left(\Omega \right)},n_{j}^{\left(\Omega \right)})$ gives the probability that site *j* is polymorphic, conditionally on the population tree and the model parameters (see the Materials and methods section).

The details of the component-wise Markov chain Monte Carlo (MCMC) algorithm, implemented in KimTree to sample from the joint posterior distribution specified by Eq (3), are provided in the Materials and Methods section. The posterior distribution of the ESR for the *i*th branch is then computed from the branch lengths at each MCMC iteration, as: $\xi_{i}=2-(9\tau_{i}^{\left(X\right)})/(8\tau_{i}^{\left(A\right)})$. Last, for each branch, we compute the support for the hypothesis *ξ*_*i*_ ≠ 0.5 as follows:

$$\begin{array}{c} S_{i}=1-2|p_{i}-0.5| \end{array}$$ (4)

where *p*_*i*_ is the proportion of the posterior MCMC draws with *ξ*_*i*_ > 0.5 in the *i*th branch. Large values of *S*_*i*_ (*S*_*i*_ → 1) are interpreted as evidence of an absence of departure from *ξ*_*i*_ = 0.5; *S*_*i*_ = 0.05 (resp. *S*_*i*_ = 0.01) indicates that 97.5% (resp. 99.5%) of the posterior MCMC draws of *ξ*_*i*_ are all larger than 0.5, or all smaller than 0.5.

### Evaluation of the model

In a preliminary evaluation, we confirmed that the improved KimTree model resulted in accurate estimates of external and internal branch lengths (see S1 Text, and S2–S5 Figs). Since the true population history is generally unknown, we investigated the power of the deviance information criterion (DIC) [35] to choose between alternative histories. To that end, we simulated 50 datasets using ms [36] for a three-population history with topology ((1,2),3). We then analyzed each of these datasets, conditionally on four alternative topologies. As in Gautier and Vitalis [32], we found that the DIC provides a clear support in favor of the true (simulated) population history (S6 Fig). We further found that, whatever the topology, the DIC supports the model where the likelihood is conditioned on SNP polymorphism (S6 Fig).

**Fig 2:**
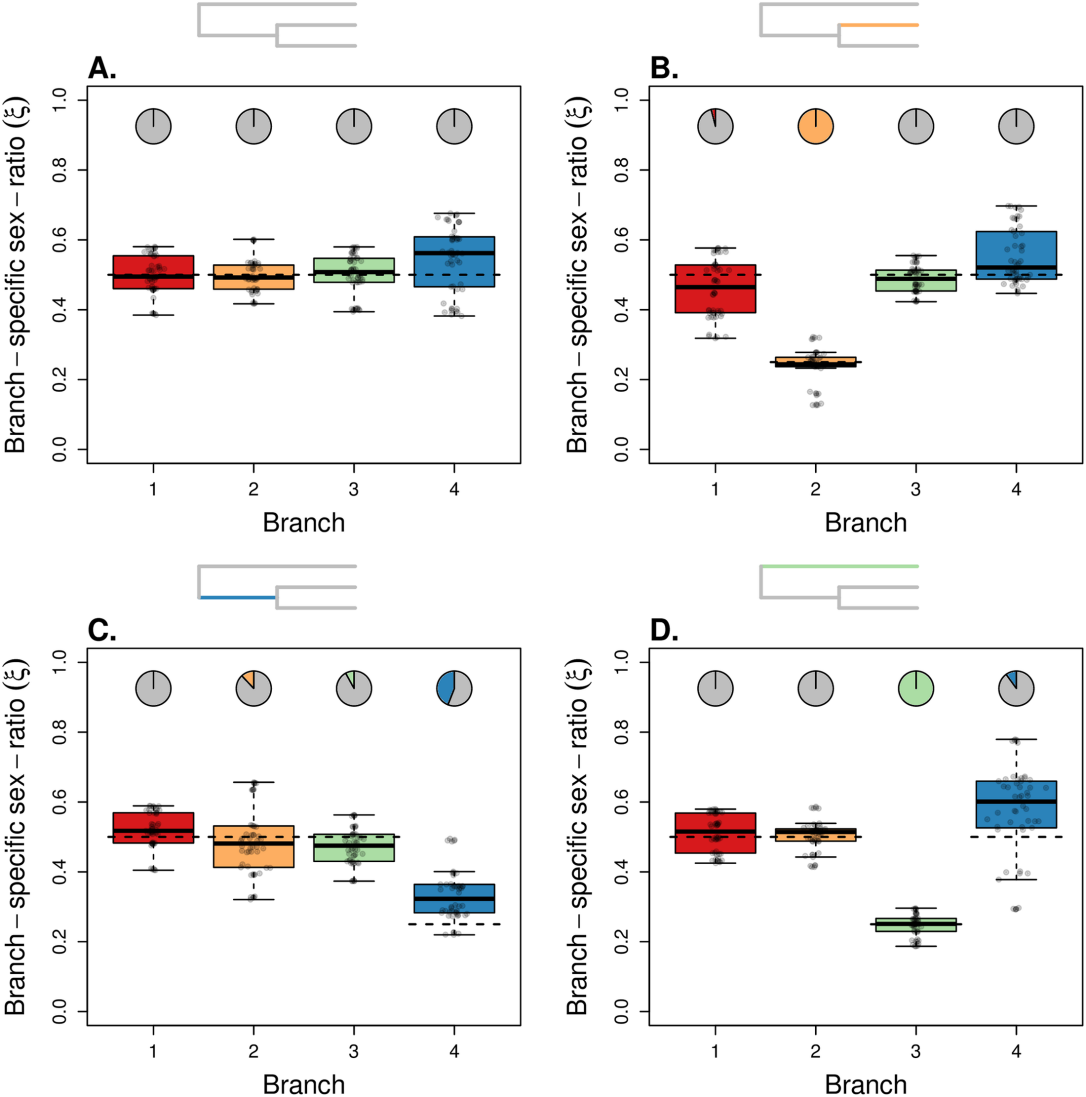
Performance of the model for estimating branch-specific sex ratios. All histories represented from A to D share the same topology ((1,2),3) but differ with respect to the simulated ESR. The root population was made of 50,000 males and 50,000 females, and each branch in the topology corresponds to a population made of 500 males and 500 females (A). In (B) branch 2 was made of 250 females and 750 males (*ξ*_2_ = 0.25); in (C) branch 4 was made of 250 females and 750 males (*ξ*_4_ = 0.25); in (D) branch 3 was made of 250 females and 750 males (*ξ*_3_ = 0.25). Inset trees indicate which branch was simulated with a biased sex ratio. The two successive splits occurred 200 and 400 generations before present time. The mutation rate was fixed at *μ* = 5 × 10^−7^. 50 females per population were sampled for each dataset. We analyzed 50 replicate simulated datasets for each scenario, with 5,000 autosomal SNPs and 5,000 X-linked SNPs. The boxplots summarize the distributions of the 50 posterior means of *ξ*_*i*_ for each of the four branches. The horizontal dashed segments indicate the true (simulated) values of *ξ*_*i*_. The pie-charts indicate the fraction of significant support values (*S* < 0.01), against the hypothesis *ξ* = 0.5 (see Eq 4).

**Fig 3:**
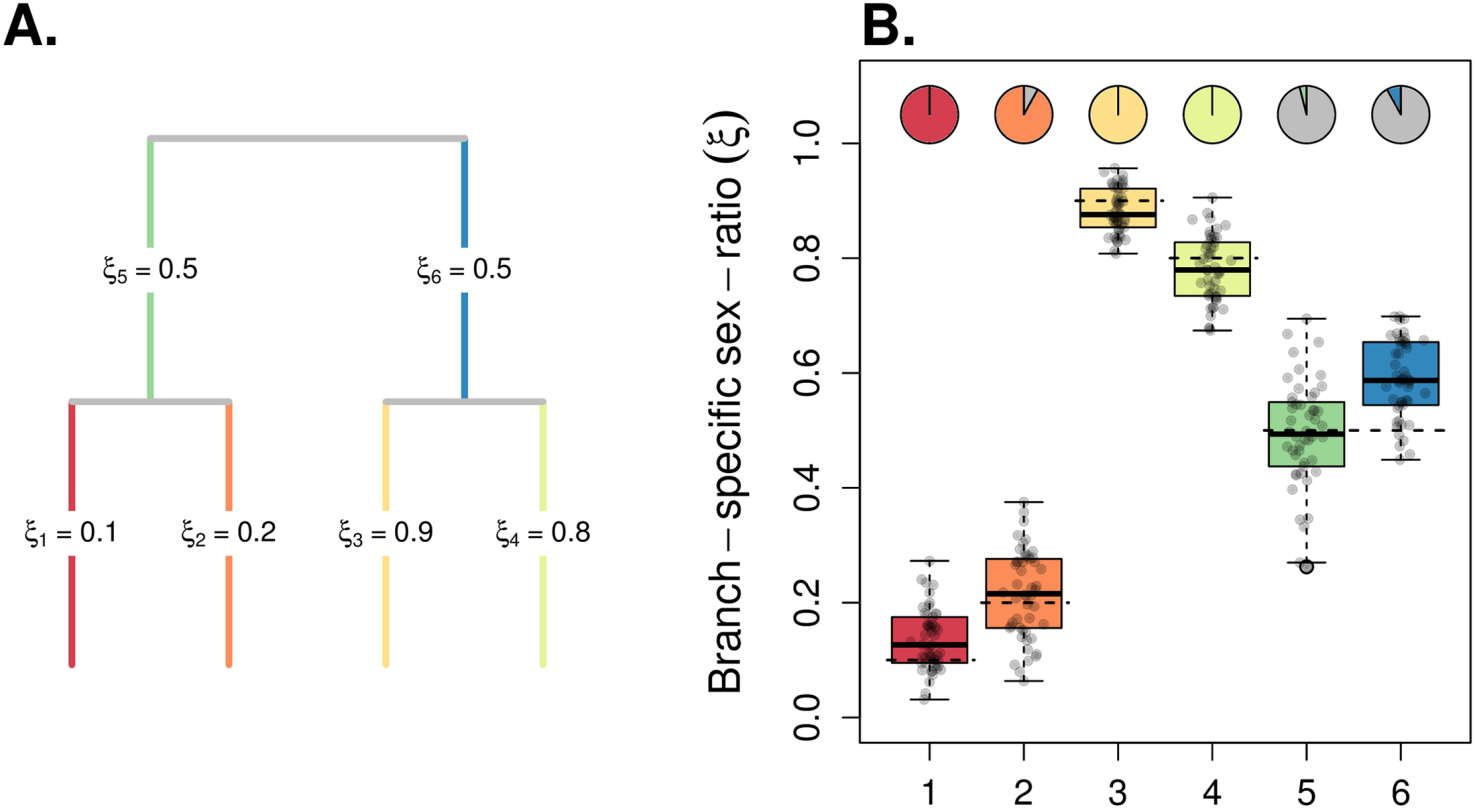
Performance of the model for estimating branch-specific sex ratios in a four-population tree. We simulated a four-population tree with topology ((1,2),(3,4)). The root population was made of 50,000 males and 50,000 females, and the internal branches correspond to populations made of 5,000 males and 5,000 females. As depicted in (A), branch 1 was made of $N_{e}^{f}$ = 1,000 females and $N_{e}^{m}=9,000$ males (*ξ*_1_ = 0.1); branch 2 was made of $N_{e}^{f}=2,000$ females and $N_{e}^{m}=8,000$ males (*ξ*_2_ = 0.2); branch 3 was made of $N_{e}^{f}=9,000$ females and $N_{e}^{m}=1,000$ males (*ξ*_3_ = 0.9); branch 4 was made of $N_{e}^{f}=8,000$ females and $N_{e}^{m}=2,000$ males (*ξ*_4_ = 0.8). The two successive splits occurred 1,000 and 3,000 generations before present time. The mutation rate was fixed at *μ* = 1.5 × 10^−7^. 50 females per population were sampled for each dataset. We analyzed 50 replicate simulated datasets of each scenario, with 5,000 autosomal SNPs and 5,000 X-linked SNPs. The boxplots in (B) summarize the distributions of the 50 posterior means of *ξ*_*i*_ for each of the six branches. The horizontal dashed segments indicate the true (simulated) values of *ξ*_*i*_. The pie-charts indicate the fraction of significant support values (*S* < 0.01), against the hypothesis *ξ* = 0.5 (see Eq 4).

**Fig 4:**
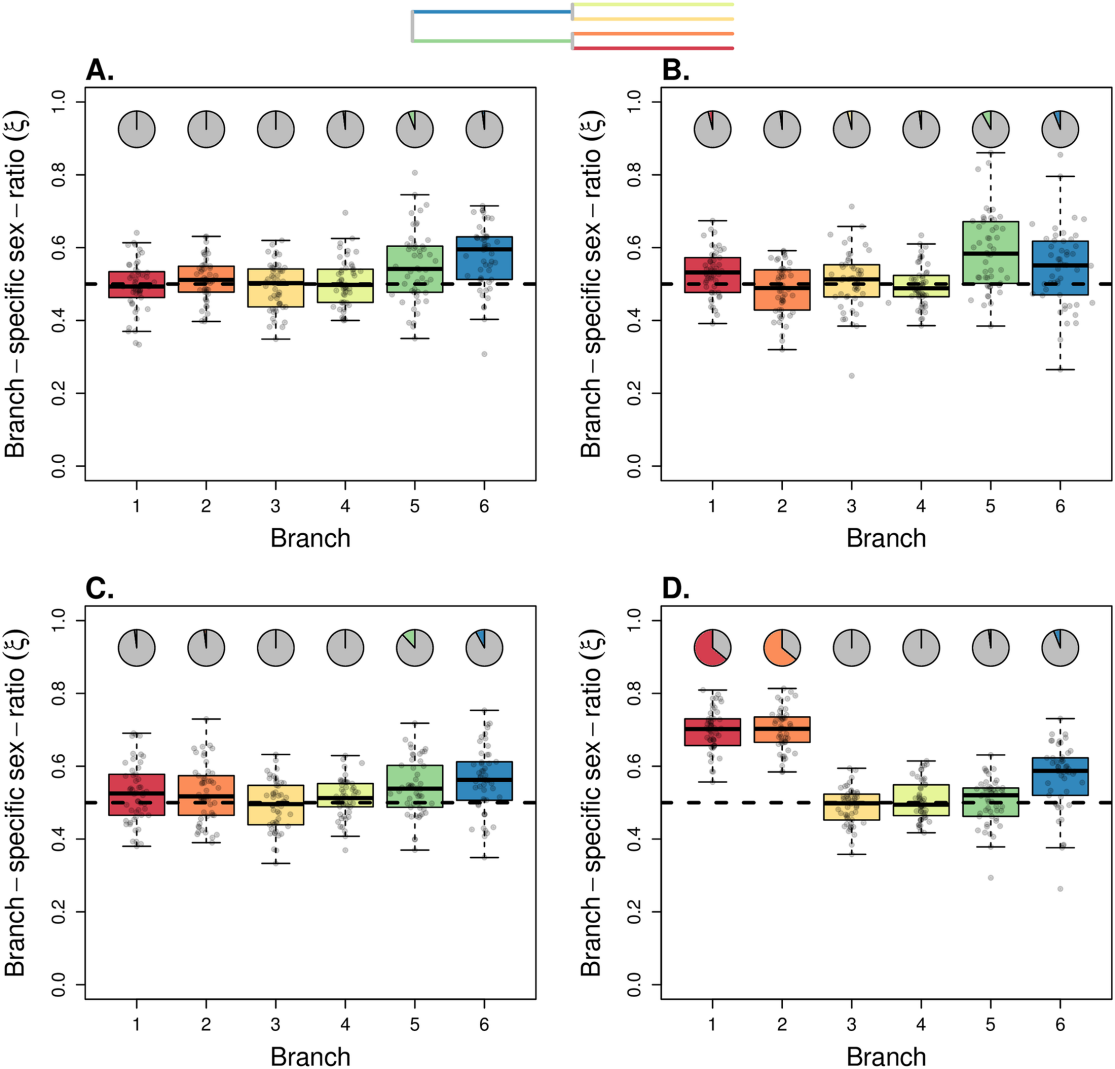
Robustness to violation of the model assumptions. We simulated four scenarios (A-D) based on a four-population tree with topology ((1,2),(3,4)), as depicted in the inset tree (top). In all scenarios, the root population was made of 50,000 males and 50,000 females, and the internal branches correspond to populations made of 5,000 males and 5,000 females. The two successive splits occurred 2,000 and 4,000 generations before present time. The mutation rate was fixed at *μ* = 1.5 × 10^−7^. 50 females per population were sampled for each dataset. In (A) the four external branches were made of $N_{e}^{f}=5,000$ females and $N_{e}^{m}=5,000$ males, and so a balanced ESR (*ξ*_*i*_ = 0.5) was assumed throughout the tree (“control” scenario). In (B), we simulated an instantaneous 5-fold population growth in branch 1 and an instantaneous 5-fold bottleneck in branch 4, both events having occurred 400 generations before present. In (C), we simulated migration between population 1 and 2, with equal rates for both sexes: *m*_f_ = *m*_m_ = 0.00025 (therefore $4N_{e}^{f}m_{f}=4N_{e}^{m}m_{m}=5$). In (D), we simulated female-biased migration between populations 1 and 2 with *m*_f_ = 0.00025 and *m*_m_ = 0 (therefore $4N_{e}^{f}m_{f}=5$ and $4N_{e}^{m}m_{m}=0$). We analyzed 50 replicate simulated datasets for each scenario, with 5,000 autosomal SNPs and 5,000 X-linked SNPs. The boxplots in (A-D) summarize the distributions of the 50 posterior means of *ξ*_*i*_ for each of the six branches. The horizontal dashed line indicates the true (simulated) values of *ξ*_*i*_. The pie-charts indicate the fraction of significant support values (*S* < 0.01), against the hypothesis *ξ* = 0.5 (see Eq 4).

Then, we evaluated the performance of our model to infer the branch-specific ESR in a population tree, using simulated datasets. First, we simulated scenarios complying to the model assumptions, with constant population sizes along each branch and no migration between branches. Since the KimTree model assumes that all polymorphisms are ancestral (an assumption which is not made in the simulations), we defined a large population size for the root population (made of 50,000 males and 50,000 females). Fig 2 shows the distributions of posterior means of branch-specific ESR, in a population tree with topology ((1,2),3), where some branches have been simulated with *ξ* ≠ 0.5. Note that an evaluation of these datasets based on wrong topologies provided consistent results for the terminal branches (see S7 Fig). Fig 3 shows a population history with topology ((1,2),(3,4)), where the four external branches have biased ESR. We found that the ESR was estimated accurately for all considered cases. Then, by altering a control case (see Fig 4A), we evaluated the robustness of our method to violations of the model assumptions.

#### Population growth, bottlenecks, and migration

We evaluated the effect of varying population size along a branch, considering population growth and bottlenecks. Fig 4B shows the distribution of posterior means for the branch-specific ESR in a population tree with topology ((1,2),(3,4)), where population 1 undergoes an instantaneous 5-fold expansion and population 4 undergoes an instantaneous 5-fold bottleneck. We found that the branch length estimates were close to their expectation (calculated using the harmonic mean of the population size along the branch), at least for the external branches (S8A and S8B Fig), and that the branch-specific ESR was not affected by population size change (S8C Fig). This result also holds for stronger (10-fold) population size changes (see S8D–S8F Fig). In general, we found our model to be robust under various ranges of population size changes.

We further tested the impact of migration between populations on inference. With equal migration rates for males and females, we estimated shorter lengths for those branches that exchange migrants. This is consistent with smaller rates of coalescence (larger effective sizes) for autosomes and X chromosome in those branches, because lineages must enter the same branch before they can coalesce. However, the estimated ESR did not deviate from the 0.5 expectation in that case (see Fig 4C). With female-biased dispersal, the branch lengths were also shorter, but the estimated ESR was biased upward (Fig 4D). This is so, because as females disperse more, X-linked lineages move more often between branches, relatively to what would occur with unbiased dispersal. This results in smaller rates of coalescence for the X chromosome, relatively to what is expected with equal migration rates for males and females [37].

#### Recent mutations

We evaluated the impact of recent mutations by varying the ancestral population size and therefore the occurrence of recent mutations in the tree (i.e., mutations occurring after the split of the root population, which are not accounted for in the KimTree model). With smaller ancestral population size, a situation that results in a more likely presence of recent mutations in the tree, we found that the internal branch lengths were largely underestimated (see S9A and S9B Fig). This result is consistent with the idea that the excess of polymorphisms within populations is (falsely) interpreted as due to larger effective sizes, and hence shorter branch lengths. The corresponding estimates for the ESR were slightly overestimated in the external branches, and more pronouncedly so in the internal branches (see S9C Fig). Sex-specific differences in mutation rates (see, e.g., [38]) could therefore possibly cause spurious signals of a biased ESR. In general, however, our simulations showed that the branch-length and ESR estimates were more accurate with larger ancestral population sizes (see S9D–S9F Fig). As it is generally the case with methods that ignore recent mutations [39], KimTree will be more accurate if populations are not strongly differentiated [32].

#### Linkage disequilibrium

With high-throughput genotyping technologies, the implicit assumption of conditional independence (i.e., exchangeability) of markers might be violated in our and other models. In particular, the correlation structure among allele frequencies at neighboring SNPs (linkage disequilibrium, LD) is not accounted for in KimTree. Furthermore, the extent of LD is expected to differ between autosomes and the X chromosome, because of the difference in effective size and the absence of recombination in males for the latter [26, 40]. We therefore tested the precision and accuracy of ESR estimates based on the analysis of linked SNPs. We found that, as expected, increasing LD between SNPs decreased the precision (but not the accuracy) of ESR estimates, which might be interpreted as the consequence of the smaller number of effectively independent markers in the data (S10 Fig). Under realistic conditions (see the “whole-genome” case in S10 Fig), however, the model was robust to LD and only slightly less precise than with truly unlinked markers.

#### Ascertainment bias

We also tested the effects of ascertaining SNPs from individuals not included in the sample (discovery panel), which may mimic datasets obtained from genotyping arrays (see Materials and methods section). To that end, we studied the effect of SNP ascertainment for a four-population tree (using the scenario from Fig 4A). To mimic ascertainment bias, we defined “ghost” individuals within some of the sampled populations, which were used only for SNP calling and discarded from further analyses. We considered three ascertainment schemes that differed by the origins of the ghost individuals used in the discovery panel (see the Materials and methods section). As shown in S11 Fig, the influence of SNP ascertainment on the estimation of branch lengths depends on the definition of the discovery panel. When all the populations contributed evenly to the discovery panel (S11A–S11C Fig), then the branch lengths for both autosomes and the X chromosome were overestimated, in particular for the internal branches. When only populations 1 and 3 contributed to the discovery panel (S11D–S11F Fig), the branch lengths of these populations were underestimated, whereas the branch lengths for populations 2 and 4 were overestimated. The estimates of internal branches showed in general the strongest deviations from the expectation. When only populations 1 and 2 contributed to the discovery panel (S11G–S11I Fig) severe biases for branch lengths were observed for the internal branches. However, in all considered ascertainment schemes, we found no evidence for a deviation from the hypothesis that *ξ* = 0.5.

#### Sample size

Although KimTree is expected to be robust to small sample sizes since it integrates over the uncertainty in population allele frequencies, it relies on a normal approximation to compute the probability of SNP polymorphism (see the Materials and methods section), which may be inaccurate when the number of lineages sampled in a population is small. Furthermore, when males are sampled, the actual sample size for X-linked markers is lower than that of autosomal SNPs. Therefore, we evaluated the robustness of KimTree to both small and unbalanced sample sizes. We found that, although the precision in ESR estimates decreases with the sample size, the accuracy is barely affected (see S12 Fig).

### Application to real data

Our simulations demonstrate that several thousand SNPs are generally sufficient to obtain accurate estimates of the model parameters. We therefore advocate for a subsampling strategy that consists in analyzing pseudo-replicated subsets of the data instead of the full data (see the Discussion section).

#### Cattle data

To test the performance of KimTree with real data, we first applied it to three different cattle breeds, namely the Holstein (HOL), Angus (ANG) and N’Dama (NDA) that are representative of various breeding schemes. The most extreme cases concern commercial dairy cattle, and to a lesser extent commercial beef breeds, here represented by HOL and ANG, respectively, where hundreds to thousands of females may be artificially inseminated with the semen of a single elite sire. In contrast, the female-bias in the ESR is expected more moderate in the traditional breeding systems of developing countries, here represented by the NDA African cattle breed, where mating is mostly uncontrolled.

**Fig 5:**
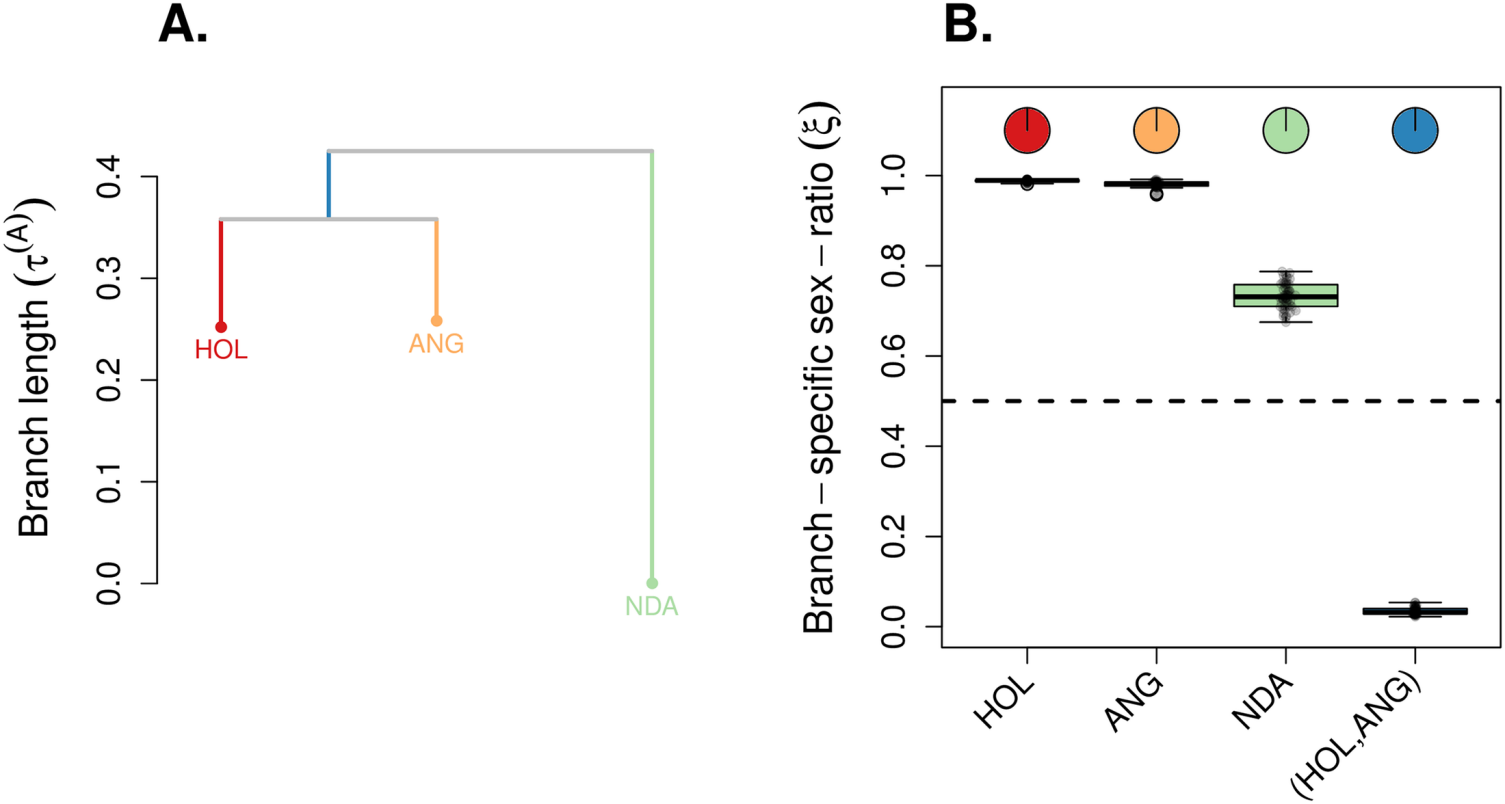
Application example on cattle data. We analyzed 643,090 autosomal SNPs and 15,009 X-linked SNPs from a dairy cattle breed (HOL), the Angus beef cattle breed (ANG), the N’Dama breed (NDA). For both genetic systems, we randomly subsampled 50 pseudo-replicated datasets from the full data, each made of 5,000 autosomal SNPs and 5,000 X-linked SNPs. We ran KimTree considering the tree topology: ((HOL,ANG),NDA) [41], represented in (A) with branch lengths estimates corresponding to the posterior means of $\tau_{i}^{\left(A\right)}\equiv t/(2N_{i}^{\left(A\right)})$. (B) The boxplots summarize the distributions of the posterior means of the ESR for each branch in the tree, for the 50 pseudo-replicated datasets. The dotted line indicates the expectation for a balanced ESR (*ξ*_*i*_ = 0.5). The pie-charts indicate the fraction of significant support values (*S* < 0.01) against the hypothesis *ξ* = 0.5 (see Eq 4).

Conditionally on the tree topology ((HOL,ANG),NDA) [41], we found that the ESR was strongly female-biased in the branches of the tree leading to HOL ($\bar{\xi }=0.988$) and ANG ($\bar{\xi }=0.981$) (see Fig 5); the ESR for NDA was found less female-biased as compared to the two commercial cattle breeds $\bar{\xi }=0.733$). However, the internal branch of the tree displayed a strongly male-biased ESR ($\bar{\xi }=0.034$). For all branches, we found a large fraction of significant support values (*S* < 0.01) against the hypothesis *ξ* = 0.5 (see Eq 4), indicating a strong support for biased ESR, independent of the direction of the bias. It should be noted that, although some individuals from African taurine populations (including NDA) were included in the discovery panel of the genotyping assay, they are under-represented compared to individuals from European origin. However, as we have previously shown (see S11 Fig), ascertainment bias is not expected to cause biased estimates of the ESR, even in the most extreme scheme, where outer branches of the population tree are not represented in the discovery panel (see S11G–S11I Fig). In such schemes, branch length estimates of the internal branch leading to the populations represented in the panel were vanishingly small (see S11G and S11H Fig). This is not what we observe from the data (see Fig 5).

#### Human data (HapMap)

**Fig 6:**
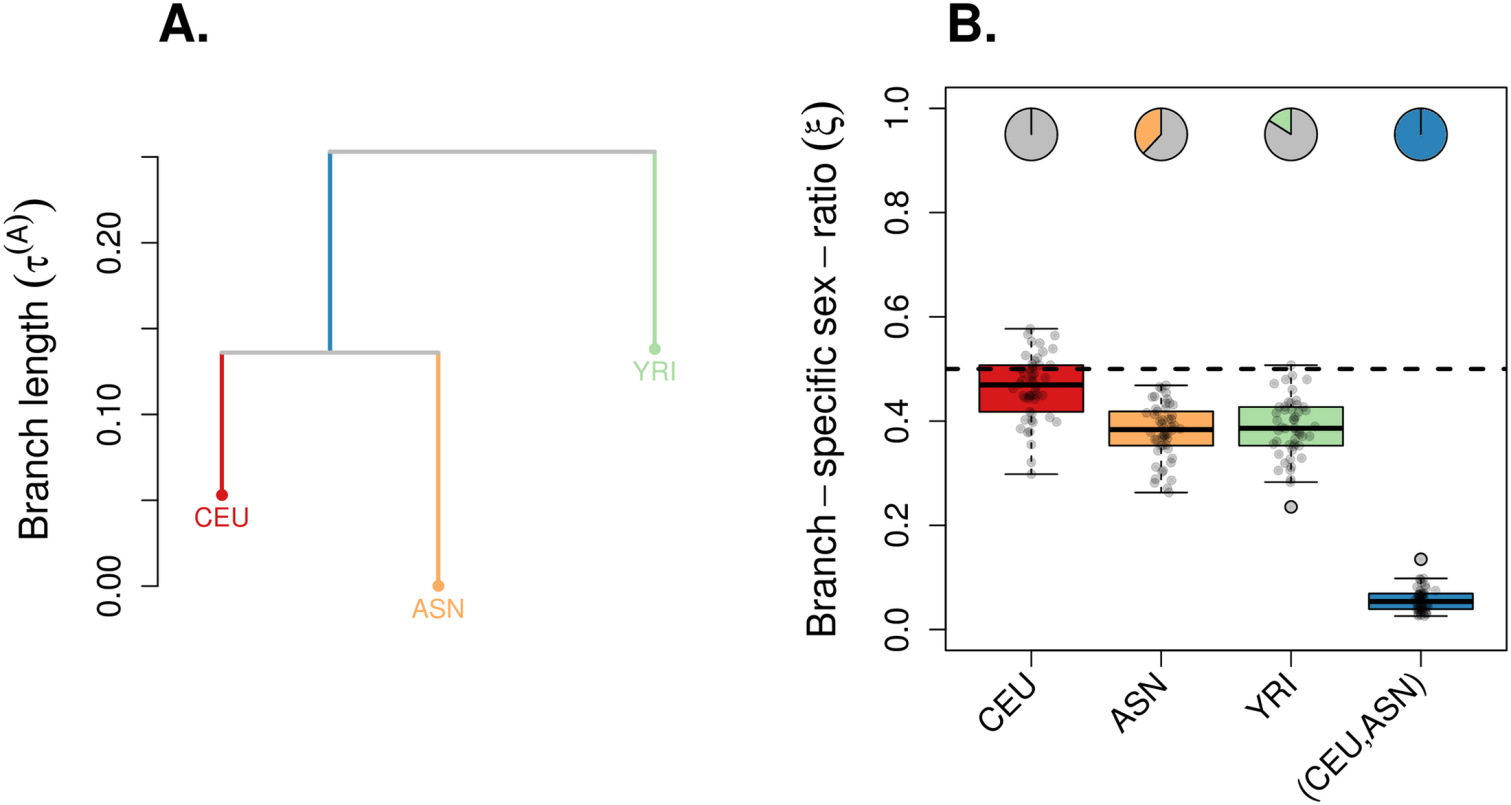
Application example on human (HapMap) data. We re-analyzed the dataset from Keinan et al. [19, 42], with genotypes from European American individuals from Utah, USA (CEU), Asian individuals grouping Han Chinese from Beijing and Japanese from Tokyo (ASN) and Yoruba individuals from Ibadan, Nigeria (YRI) (see the Materials and methods section). The data consisted of 340,909 autosomal SNPs and 12,737 X-linked SNPs. For both genetic systems, we randomly subsampled 50 pseudo-replicated datasets from the full data, each made of 5,000 autosomal SNPs and 5,000 X-linked SNPs. We ran KimTree conditionally on the ((CEU,ASN),YRI) topology, represented in (A) with branch lengths estimates corresponding to the posterior means of $\tau_{i}^{\left(A\right)}\equiv t/(2N_{i}^{\left(A\right)})$. (B) The boxplots summarize the distributions of the posterior means of the ESR for each branch in the tree, for the 50 pseudo-replicated datasets. The dotted line indicates the expectation for a balanced ESR (*ξ*_*i*_ = 0.5). The pie-charts indicate the fraction of significant support values (*S* < 0.01) against the hypothesis *ξ* = 0.5 (see Eq 4).

We re-analyzed the dataset from Keinan et al. [19, 42], with genotypes from European American individuals from Utah, USA (CEU), Asian individuals grouping Han Chinese from Beijing and Japanese from Tokyo (ASN) and Yoruba individuals from Ibadan, Nigeria (YRI) (see the Materials and methods section). We ran KimTree conditionally on the ((CEU,ASN),YRI) tree topology and found no evidence for a severe deviation from a balanced ESR in Europeans, Asians and Africans. However, the internal branch, ancestral to Europeans and Asians, showed a strongly male-biased ESR (see Fig 6), consistent with the results of the original analyses by Keinan et al. [19]. It is worth noting that the conclusions raised by Keinan et al. [19] were based on an extrapolation from independent analyses of pairs of populations combined with information from the site frequency spectrum, instead of a joint analysis of the three populations altogether, as we have done here. We could however also reproduce their results by running independent, pairwise KimTree analyses. Consistently, we found little bias in ESR for both CEU and ASN, when analyzed together, but a male-biased ESR when CEU and ASN were compared with YRI (see S13 Fig).

#### Human data (whole-genome sequence)

**Fig 7:**
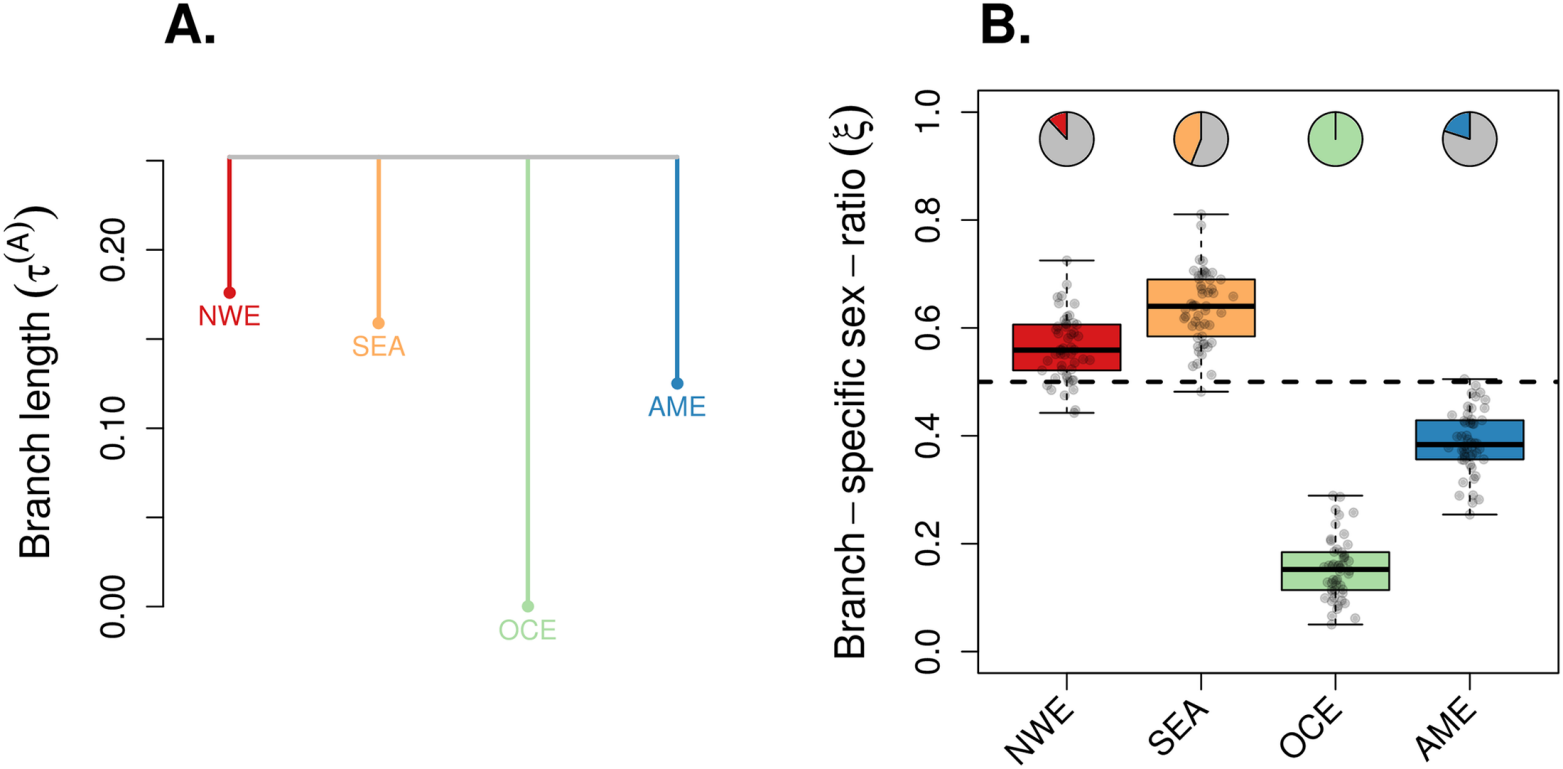
Application example on whole-genome human sequence data. We re-analyzed a subset of the whole-genome sequence data from Pagani et al. [33], with populations from NW-Europe (NWE), SE-Asia (SEA), Oceania (OCE) and Americas (AME) (see the Materials and methods section for a detailed composition of populations). For both genetic systems, we randomly subsampled 50 pseudo-replicated datasets from the full data, each made of 5,000 autosomal SNPs and 5,000 X-linked SNPs. We ran KimTree considering the best fitting tree topology (NWE,SEA,OCE,AME) (see the Materials and methods section), represented in (A) with branch lengths estimates corresponding to the posterior means of $\tau_{i}^{\left(A\right)}\equiv t/(2N_{i}^{\left(A\right)})$. (B) The boxplots summarize the distributions of the posterior means of the ESR for each branch in the tree, for the 50 pseudo-replicated datasets. The dotted line indicates the expectation for a balanced ESR (*ξ*_*i*_ = 0.5). The pie-charts indicate the fraction of significant support values (*S* < 0.01) against the hypothesis *ξ* = 0.5 (see Eq 4).

Finally, we used KimTree to re-analyze a subset of the whole-genome sequence data from Pagani et al. [33], which should minimize SNP ascertainment bias. We ran KimTree considering the best fitting tree topology (NWE,SEA,OCE,AME) (see the Materials and methods section), which is consistent with a rapid split of all the sampled populations from their common recent ancestor (see Fig 7A). We estimated a long autosomal branch length for Oceania (${\bar{\tau }}_{\text{OCE}}=0.252$), as compared to the other populations in Asia (${\bar{\tau }}_{\text{SEA}}=0.093$), Europe (${\bar{\tau }}_{\text{NWE}}=0.076$), and the Americas (${\bar{\tau }}_{\text{AME}}=0.127$). We found that the ESR in the Americas shows no support for a deviation from 0.5; we further found that the ESR for NW-Europeans and SE-Asians show some support for a moderate deviation from 0.5; in Oceania, we found a large support for a strongly male-biased ESR (see Fig 7B). Since the Oceanian sample consisted of only six males, we analyzed simulated datasets mimicking these human data, using the same topology, sample sizes, and estimated autosomal branch lengths, but assuming a balanced ESR in all branches. We found no support for a deviation of the ESR from 0.5, that would have been caused by a small sample size in the Oceania-like branch (S14 Fig).

## Discussion

In this study, we introduced an improved and extended KimTree model that can be used to infer branch lengths and branch-specific ESR for a given tree topology, taking advantage of a joint analysis of X-linked and autosomal allele frequency data.

The inference of branch-specific ESR throughout a population tree requires accurate estimates of branch lengths from autosomes and X chromosome. Model-based methods that reconstruct population histories can be broadly divided into two categories: coalescent-based models (e.g., [43]) and models that use diffusion approximations of genetic drift (e.g., [44]). However, despite considerable computational advances, coalescent-based likelihood inferences remain in practice intractable when the size of the considered data is large [43, 45]. Recently, Tataru et al. [46] evaluated the accuracy of Kimura’s time-dependent diffusion approximation for genetic drift, relatively to alternative models like the Gaussian (used in, e.g., TreeMix [39]), the beta distribution (used in, e.g., NB [47]) or the beta with spikes approximation (used in SpikeyTree [48]). As expected, they found that Kimura’s time-dependent diffusion provides the most accurate approximation to the drift process. Yet, for branch length inference, Tataru et al. [48] showed that SpikeyTree could outperform KimTree [32], which is based on Kimura’s time-dependent diffusion. We have shown that this discrepancy originated from the fact that in its original implementation, KimTree did not account for the exclusive presence of polymorphic markers in SNP datasets. By construction, these datasets lack the information contained in the fraction of sites that are polymorphic in the root population, but fixed in the sample (see S2 and S3 Figs). Following Tataru et al. [48], we therefore extended our model to condition on polymorphism at all sites. When compared to the full likelihood model, this conditional likelihood model is strongly supported, based on the DIC criterion (S6 Fig). We have shown that branch length estimates were improved, particularly for internal branches. In a direct comparison, the improved KimTree model outperformed the beta-with-spikes model [48] (see S4 and S5 Figs).

We demonstrated through extensive simulations that our method is able to accurately infer the ESR for different scenarios, if the model assumptions are met (Figs 2 and 3). However, as the ESR is known to be affected by different processes such as selection [49–51], sex-biased migration [52], population size changes [53] or SNP ascertainment bias, it is necessary to interpret the results with care. Furthermore, it should be noted that our model cannot distinguish between possible sources of variation for the ESR. For example, social organization (polygamy), sex-specific migration, or differential mortality rates may lead to a similarly unbalanced ESR. Thus, any of such mutually non-exclusive alternatives must be considered when interpreting the results. Independent analyses might therefore be helpful. For instance, computing *f*-statistics [54, 55] may serve as a sanity check to rule out substantial migration among populations.

However, we have shown that our parameter estimates are robust to different model violations (Fig 4 and S8 Fig). In general, estimates of the ESR for external branches seem to be more robust than estimates for internal branches. This might be due to a higher power in characterizing recent ESR as compared to ancestral ones. In addition, recent (non-ancestral) polymorphism seems to more strongly affect internal branches, possibly contributing to a higher uncertainty in the ESR for those branches (S9 Fig). Population size changes may alter the X-to-autosome pattern of diversity [53], which can then lead to biased estimates of the ESR. The reason for this is the smaller effective population size of the X chromosome compared to the autosomes, allowing X-linked variation to converge faster to its new equilibrium after a population size change. With our approach, we found no evidence for a bias in estimating the ESR due to population size changes: each branch length estimate is very close to that predicted using the harmonic mean of the effective size along that branch, such that the corresponding ESR appears unbiased (Fig 4B and S8 Fig).

Although the assumption of conditional independence of SNPs is violated in KimTree, and although the expected extent of LD differs between autosomes and the X chromosome, we found that our model is robust to LD under realistic conditions (S10 Fig). Based on our simulation results, we therefore recommend to subsample SNPs randomly, or to thin the data by taking one SNP out of every *n* SNPs from the ordered map. Such a strategy is more relevant than LD pruning, because it does not alter the allele frequency spectrum, on which inference is based. Random subsampling of genome-wide data can further be used to provide pseudo-replicated estimates from a handful of reduced datasets. This allows in turn to provide higher support to our conclusions through pseudo-independent estimates of the parameters of interest. From a more technical point of view, another advantage of this approach is that we may reduce the asymmetry in the number of markers for autosomes and the X chromosome. This asymmetry in the amount of information available for each genetic system may indeed cause specific issues for the joint update of branch lengths, with poor acceptance rates. We found that 5,000 markers per dataset and per genetic system provided accurate parameter estimates, while limiting the computational burden.

Estimation of the ESR might also be affected by SNP ascertainment bias, which notably depends on the ascertainment scheme. Although conditioning the likelihood on the presence of polymorphic sites only does improve the accuracy of branch length estimates (see above), it does not address the specific problem of ascertainment bias in genotyping assays. We found that ascertainment bias may result in biased estimates of branch lengths, in particular when only a subset of populations belongs to the discovery panel (see S11A, S11B, S11D, S11E, S11G and S11H Fig). However, estimates of the ESR were unbiased in the simulated conditions, where the ascertainment scheme was identical for both autosomal and X-linked markers (see S11C, S11F and S11I Fig). Nevertheless, we recommend to be cautious when interpreting the results from ascertained datasets and, if possible, to use whole-genome sequence data.

For illustration purposes, we analyzed both cattle and human SNP genotyping data, providing new insights into the sex-specific demographic history of these two species. We chose three cattle breeds (HOL, ANG and NDA) with contrasting breeding schemes (from a widespread use of artificial insemination in the HOL dairy cattle to mostly uncontrolled mating in the NDA cattle from West-Africa). These breeds are also representative of the post-domestication history, with HOL, ANG and NDA presumably originating from the same domestication center in the Middle East, ca. 10,000 YBP [56]. As expected, we found a strongly female-biased ESR in the commercial breeds (HOL and ANG), with less than two effective males for 100 effective females in both breeds. These ESR estimates integrate over the time of divergence between ANG and HOL, which has occurred ca. 2,000 YBP [57]. Since modern genetic improvement programs have been generalized only recently (in the past 70 years), the impact of increased selective pressure for beef (in ANG) or milk (in HOL) production on the ESR might thus be even higher than our estimate suggests. Before that, indeed, the ESR for commercial cattle breeds might have been only moderately female-biased, as we observe for the traditionally raised NDA with about 36 effective males for 100 effective females. More interestingly, we found a strongly male-biased ESR (four effective females for 100 effective males) in the internal branch of the tree, which is ancestral to the ANG and HOL breeds. This result supports the hypothesis that around the period of cattle domestication, females were plausibly more easily managed than males. Keeping and rearing preferentially female offspring would indeed tend to decrease the effective size for females. At the same time, preventing tamed females from breeding randomly with wild males would be a difficult task, which would result in turn in an increased effective size for males (see [58], p. 2218), and therefore in a male-biased ESR. Alternatively, introgression of wild auroch males into domesticated cattle [59, 60] may have increased the male effective population size. Deciphering between these two non-mutually exclusive hypotheses would require further investigations.

Finally, we re-analyzed recently published sequence data from Pagani et al. [33] combined with sequences from Drmanac et al. [61] and from the Personal Genomes Project. We found a strong and significant male-biased ESR in the Oceanian sample (Fig 7), that could not be explained by the small sample size in that branch (S14 Fig). It should be pointed out, however, that because this Oceanian sample consists of only six males, it may not be representative for the whole region. Nevertheless, our results are consistent with Malaspinas et al. [4], who recently studied high-coverage genomes in a large dataset from Aboriginal Australians and Papuans and provided important insight into the social structure of Aboriginal Australian societies. They inferred greater between-group variation for mtDNA compared to the Y chromosome, suggesting higher levels of male-biased dispersal. The lack of recombination in these markers, however, may complicate the interpretation of their observed patterns of genetic diversity [15, 16]. With our new approach, we provide additonal evidence of a male-biased ESR in Oceanians, here on the basis of autosomal and X-linked data, which take advantage of thousands of independent loci. Combining these results strengthens the picture of complex marriage and post marital residence patterns among Pama-Nyungan Australian groups, where tribes are divided into exogamous “sections” that are either patrilineal or matrilineal [62]. Matrilineal organization should increase relatedness among women, and therefore reduce the effective number of women as compared to men, which may result in a male-biased effective sex ratio, as we observed.

Our method takes advantage of genome-wide SNP data and can in principle be applied to a wide range of species. Its generic character allows it to be also applicable to Pool-seq data, which in contrast to individual sequencing, is based on sequencing individuals in pools, resulting in read count data instead of individual genotypes. Pool-seq allows for cost efficient production of large datasets, and recently became a popular source of data due to its high accuracy-to-cost ratio [63]. For Pool-seq data, one shall assume that the (observed) read counts are binomially distributed, given the (unknown) allele frequencies and the sample size of each pool [64], which is straightforward to implement in our hierarchical Bayesian framework [34]. It should be noted however that conditioning the likelihood on the exclusive presence of polymorphic sites in the sample has to be further adjusted for Pool-seq data. Although sites that are fixed among all sampled individuals are also fixed in the Pool-seq data (baring mutation), it may happen that polymorphic sites among sampled individuals appear fixed in the Pool-seq data (if, by chance, only one allele is sequenced in the Pool-seq experiment). This latter possibility must therefore be accounted for when calculating the probability of a polymorphic site in the case of Pool-seq data.

Moreover, our method can in principle also be used to detect selection by identifying outliers on either autosomes or X chromosome. This can be achieved by computing (locus-specific) posterior predictive *p*-values, to test if the observed data are plausible under the posterior predictive distribution [65, 66]. With our model, we can take advantage of the relationship between autosomes and X chromosomes via the ESR and, for example, test for signatures of selection on the X chromosome, while accounting for the demographic information contained in autosomal data. Such an approach was suggested by Dutheil et al. [67], who analyzed whole-genome data of humans and great apes. They used autosomal data to predict the expected incomplete lineage sorting for the X chromosome, assuming a balanced sex ratio, and found evidence for recurrent selective sweeps on the X chromosome. Using KimTree, we may similarly infer demographic parameters (branch lengths and branch-specific ESR) from the joint analysis of autosomal and X-linked markers, and test for locus-specific departures of that demographic history, which might result from selection acting on either genetic system.

## Materials and methods

### Conditioning on polymorphic sites

Because SNP data from different populations contain, by definition, only polymorphic sites, we condition the likelihood to account for those sites that are polymorphic in the root population but end up as fixed positions in the full sample and are, as such, absent from the dataset (see Tataru et al. [48]). In the following, for the sake of clarity, we develop the computation of the conditional likelihood in the context of the simpler model defined by Eq (1). This computation extends naturally to the full model defined by Eq (3), for both autosomal and X-linked data. Conditioning the likelihood amounts to defining an indicator variable λ_*j*_, which equals 1 if the *j*th position is polymorphic in the full sample (i.e., if 0 < ∑_*i*_ *y*_*ij*_ < ∑_*i*_ *n*_*ij*_). As detailed below, we assume that the prior on λ_*j*_ depends on the sample size **n**_*j*_, the branch lengths ***τ*** and the allele frequencies in the root population *x*_*rj*_:

$$\begin{array}{ccc} P(\lambda_{j}=1|n_{j},x_{rj},\tau )=1 & - & P(\sum_{i}y_{ij}=0|n_{j},x_{rj},\tau )\\ & - & P(\sum_{i}y_{ij}=\sum_{i}n_{ij}|n_{j},x_{rj},\tau ) \end{array}$$ (5)

where $P(\sum_{i}y_{ij}=0|n_{j},x_{rj},\tau )$ is the probability that the reference allele is absent in all sampled populations and likewise $P(\sum_{i}y_{ij}=\sum_{i}n_{ij}|n_{j},x_{rj},\tau )$ is the probability that the reference allele is fixed in the entire sample. Altogether, the conditional probability of the data (likelihood) therefore reads:

$$\begin{array}{ccc} \pi (Y|N,X,\tau ,\alpha ,\beta ,\lambda =1) & \propto & \frac{\pi (Y,\lambda =1|N,X,\tau ,\alpha ,\beta )}{\prod_{j=1}^{J}P\left(\lambda_{j}=1|n_{j},x_{rj},\tau \right)}\\ & \propto & \frac{\pi (Y|N,X)\pi (X|\tau ,\alpha ,\beta )}{\prod_{j=1}^{J}P\left(\lambda_{j}=1|n_{j},x_{rj},\tau \right)} \end{array}$$ (6)

In order to develop Eq (5), we suggest an approach based on coalescent theory, similar in spirit to that described in Beaumont [68]. In a single population (or a branch in a population tree), the number of ancestral lineages of a sample of genes decreases over time (looking backward) due to coalescent events. Therefore, in the absence of newly arising mutations, the *j*th site will be fixed in the sampled populations, if all the ancestral lineages of the sample in the root node carry the same allelic state, i.e. $P\left(\sum_{i}y_{ij}=0\right)=P\left(y_{rj}=0\right)$ and $P\left(\sum_{i}y_{ij}=\sum_{i}n_{ij}\right)=P\left(y_{rj}=n_{rj}\right)$. The probabilities $P(y_{rj}=0)$ and $P(y_{rj}=n_{rj})$ may be obtained by integrating over the probability distribution of the number of ancestral lineages in the root node, weighted by the probability that all the ancestral lineages are of the same allelic type (see below).

The number of ancestral lineages in the root node, which is a random variable, depends upon the number of coalescences that occur in the intervals between the nodes of the tree. For each interval (i.e., for each branch), we therefore need to compute the number of ancestral lineages, looking backward in time, given the current number of lineages and the branch length. Tavaré [69] derived the distribution of the number *k* of ancestral lineages P(k∣i,τ) for one population, given the current number of lineages *i*, and the time interval *τ* (in a diffusion time-scale). Because computation of Tavaré’s [69] distribution was shown to be unstable [70, 71], we use instead a normal distribution approximation to P(k∣i,τ) (see Eqs 4 and 5 in Griffiths [70]).

To integrate over the full population tree, we start the computation at the leaf nodes, where the number of lineages equals the corresponding sample size *n*_*ij*_ (measured in numbers of genes), i.e. we compute $P({\tilde{n}}_{a(i)j}|n_{ij},\tau_{i})$ for *i* = 1, …, *I* using Eqs (4) and (5) in Griffiths [70]. Here, ${\tilde{n}}_{a(i)j}$ is the (random) number of lineages in the ancestral node *a*(*i*) of *i*. We then proceed towards the root of the tree by computing $P({\tilde{n}}_{a(i)j}|{\tilde{n}}_{ij},\tau_{i})$ for all internal nodes, i.e. for *i* = *I* + 1, …, *r*.

For each internal node, we first need to compute the probability distributions of the number of lineages $P_{c}\left({\tilde{n}}_{a(i)j}\right)$, which is a combination of the probability distributions of the number of lineages for all the daughter nodes of *a*(*i*). For example, in the case of two nodes *i* and *i*′ that share the same ancestor, i.e. *a*(*i*) = *a*(*i*′), we get the following probability distribution:

$$\begin{array}{c} P_{c}\left({\tilde{n}}_{a(i)j}=k\right)=\sum_{l=1}^{n_{ij}}\sum_{m=1}^{n_{i^{\prime }j}}P\left(l|n_{ij},\tau_{i}\right)P\left(m|n_{i^{\prime }j},\tau_{i^{\prime }}\right)\delta_{k}^{l+m} \end{array}$$ (7)

where $\delta_{k}^{l+m}$ is the Kronecker delta:

$$\begin{array}{c} \delta_{k}^{l+m}=\{\begin{array}{c} 1\;\text{if}\;k=l+m\\ 0\;\text{otherwise}. \end{array} \end{array}$$ (8)

Note that, in general, different combinations of *l* and *m* contribute to the probability of a single number of lineages *k* = *l* + *m*. Also, note that the probability distribution $P_{c}\left({\tilde{n}}_{a(i)j}\right)$ for the number of ancestral lineages in that node is defined for *k* = 2, …, (*n*_*ij*_ + *n*_*i*′*j*_) lineages (*k* = 2 because the node *a*(*i*) has two daughter nodes in that example). The case of more than two populations sharing the same ancestral node follows analogously. The full probability distribution of ancestral lineages for the node *a*(*i*) after time *τ*_*a*(*i*)_ is then be given by:

$$\begin{array}{c} P\left({\tilde{n}}_{a(i)j}=k^{\prime }|\tau_{a(i)}\right)=\sum_{k}P\left(k^{\prime }|k,\tau_{a(i)}\right)P_{c}\left({\tilde{n}}_{a(i)j}=k\right) \end{array}$$ (9)

Combining all branches, recursively, in the population tree, we get the probability distribution of the number of ancestral lineages in the root node *r* at site *j*, $P({\tilde{n}}_{rj}|\tau )$. Given that the allele frequency in the root population at site *j* is *x*_*rj*_, we get:

$$\begin{array}{c} P\left(y_{rj}=0|x_{rj},n_{j},\tau \right)=\sum_{k}P\left({\tilde{n}}_{rj}=k|n_{j},\tau \right){\left(1-x_{rj}\right)}^{k} \end{array}$$ (10)

and:

$$\begin{array}{c} P\left(y_{rj}=n_{rj}|x_{rj},n_{j},\tau \right)=\sum_{k}P\left({\tilde{n}}_{rj}=k|n_{j},\tau \right)x_{rj}^{k} \end{array}$$ (11)

Therefore, combining Eqs (5), (10) and (11), the probability that all the ancestral lineages in the root node are not of the same allelic type (and therefore that the full sample is polymorphic) is given by:

$$\begin{array}{c} P\left(\lambda_{j}=1|x_{rj},n_{j},\tau \right)=1-[\sum_{k}P\left({\tilde{n}}_{rj}=k|n_{j},\tau \right)[{\left(1-x_{rj}\right)}^{k}+x_{rj}^{k}]] \end{array}$$ (12)

For ease of computation, we assume the same sample size **n** across all sites, which we set to the maximum sample size observed in the dataset. Then the number of ancestral lineages in the root node, $P({\tilde{n}}_{rj}=k|n,\tau )$, is independent of site *j* and is therefore equal across loci.

Since the probability of a site to be polymorphic is conditioned on the allele frequency in the root population (*x*_*rj*_), the beta distribution for the allele frequencies in the root population must be interpreted as the distribution of allele frequencies only for sites that are polymorphic in the entire sample. This is different from the model by Tataru et al. [48], who instead computed the probability of a site to be polymorphic by integrating over the beta distribution of allele frequencies in the root population (with shape parameters *α* and *β*). In their case, the beta distribution therefore corresponds to the distribution of allele frequencies in the root population, i.e., not only for polymorphic sites but also for sites that were polymorphic in the root population and became fixed in the entire sample. In practice, we found both implementations (and therefore both interpretations of the beta distribution) to result in similar estimates for the branch lengths. However, integrating over the beta distribution, as in Tataru et al. [48], sometimes resulted in numerical issues related to the computation of the hyper-parameters *α* and *β*, which convinced us that this approach was less robust. Consequently, all the results presented here are based on computing the probability of a site to be polymorphic conditionally on the allele frequencies (*x*_*rj*_) in the root population.

### Implementation

We implemented a component-wise Markov chain Monte Carlo (MCMC), or Metropolis within Gibbs, algorithm (see, e.g., [72]) to sample from the joint posterior distribution of π(Θ,λ=1∣D), which is specified by Eq (3). For all parameters but $\tau_{i}^{\left(A\right)}$ and $\tau_{i}^{\left(X\right)}$, this amounts to updating one parameter at each step, iteratively, as detailed in Gautier and Vitalis [32]. For the branch lengths, however, we perform a joint update for $\tau_{i}^{\left(A\right)}$ and $\tau_{i}^{\left(X\right)}$, assuming a bivariate uniform prior distribution over the support that satisfies $9\tau_{i}^{\left(X\right)}/16<\tau_{i}^{\left(A\right)}<9\tau_{i}^{\left(X\right)}/8$ and $8\tau_{i}^{\left(A\right)}/9<\tau_{i}^{\left(X\right)}<16\tau_{i}^{\left(A\right)}/9$ (see S1 Fig). At each step of the Markov chain, and for each branch, a new value of $\tau_{i}^{\left(A\right)}$ is drawn from a uniform distribution centered around the current value; if the proposed value lies outside the support defined above, then the excess is reflected back into the support. The same procedure is executed for $\tau_{i}^{\left(X\right)}$, and the update is accepted or rejected for both parameters altogether, using appropriate Metropolis-Hastings ratios.

The proposal distributions for each of the **X**^(Ω)^, ***τ***^(Ω)^, *α*^(Ω)^ and *β*^(Ω)^ parameters are adjusted by means of short pilot runs (typically 20 runs with 500 iterations), executed before the MCMC, to obtain acceptance rates between 0.25 and 0.40 (see, e.g., [73]). Under default conditions, each MCMC was run for 20,000 iterations after a burnin-in period of 10,000 runs. Samples from the posterior distribution were taken every 20 iterations (thinning) to reduce autocorrelation.

### Model assessment

Because the tree topology is generally unknown, we implemented a model choice procedure to characterize, for any given dataset, the strength of evidence for alternative population histories. Following Gautier and Vitalis [32], we used the deviance information criterion (DIC), which is a standard criterion for model selection [35]. Up to a constant that does not depend on the model, the DIC is equal to $\left(2\bar{D}-D\left(\bar{\Theta }\right)\right)$, where $\bar{D}$ is the posterior mean deviance, which can be interpreted as a Bayesian measure of fit, and $D(\bar{\Theta })$ is the Bayesian deviance evaluated at the posterior mean of the parameters **Θ**. Extending Eq (8) from Gautier and Vitalis [32] to our model gives (dropping the index Ω for the sake of clarity):

D¯=-2T∑t=1T[∑i=1I∑j=1Jlog[(nijyij)xij(t)yij(1-xij(t))nij-yij]-∑j=1JlogP(λj=1∣nj(t),xrj(t),τ(t))] (13)

and:

D(θ¯)=-2[∑i=1I∑j=1Jlog[(nijyij)x¯ijyij(1-x¯ij)nij-yij]-∑j=1JlogP(λj=1∣nj,x¯rj,τ¯)] (14)

In Eq (13), *x*_*ij*_(*t*) is the *t*th sampled value of the parameter *x*_*ij*_ along the MCMC, out of *T* total draws. In Eq (14), ${\bar{x}}_{ij}=\frac{1}{T}\sum_{t=1}^{T}x_{ij}\left(t\right)$ is the posterior mean of *x*_*ij*_, and $\bar{\tau }$ is the vector of the posterior means of the branch lengths.

### Simulations

To evaluate the performance of our model to estimate the ESR from autosomal and X-linked data, we used a generation-by-generation coalescent based simulator [74]. In brief, the simulator is based on an algorithm in which coalescence and migration events are considered generation-by-generation until the common ancestor of the whole sample is reached (see, e.g., [75]). This simulator allows us to specify male and female effective population sizes, and sex-specific migration rates, for each branch in a population tree for any defined demography. The algorithm also accounts for the specificities of autosomal and X chromosomal patterns of inheritance. All loci are simulated strictly independently (no pedigree is constructed during the simulations, and coalescent trees are therefore independent across loci). Each locus is constrained to be strictly bi-allelic (i.e., all coalescent trees with more than a single mutation are discarded). The mutation rate was set to *μ* = 1.5 × 10^−7^ with an ancestral (root) population made of 50,000 males and 50,000 females. In general, we simulated 5,000 autosomal markers and 5,000 bi-allelic X-linked markers. We sampled 50 diploid females from each population (such that the number of sampled genes is 100 for both autosomal and X-linked markers). Typically, 50 independent datasets were simulated for each scenario.

The analysis of SNP data is intricate due to the discovery protocols used to ascertain polymorphisms. Typically, SNPs are called using genotypes from a reduced sample of individuals, which is referred to as the discovery panel. Only then, the ascertained SNPs are genotyped in the full sample of interest. As a consequence, the data contain less low-frequency alleles than expected in the absence of ascertainment [76]. To analyze the consequences of SNP ascertainment bias on the inference of the ESR, we simulated SNP datasets mimicking different ascertainment schemes. For all schemes, we considered a population history with balanced topology ((1,2),(3,4)). We called SNPs using two “ghost” individuals (out of 50 simulated diploid females) in a panel of populations. These individuals were used exclusively for SNP calling and discarded from further analyses. Only those sites that were polymorphic in the discovery panel were therefore considered for the KimTree analyses, using allele counts from the remaining 48 individuals of each sample. We considered three schemes differing by the populations contributing to the panel. In the first scheme, all populations (1–4) were represented in the discovery panel. In the second scheme, only populations 1 and 3 (that belong to both sides of the balanced tree) were represented in the panel. In the third scheme, only populations 1 and 2 (that belong to a single side of the balanced tree) were represented in the panel.

To evaluate the robustness of the model to LD, we simulated additional datasets using msprime [77], because our generation-by-generation simulator is not designed to generate linked markers. Considering a population history with balanced topology ((1,2),(3,4)), we generated 100 haplotypes of 100 Mb (1 Morgan in our parameterization) for each population and each genetic system. Assuming a balanced ESR, we considered *N*_e_ = 1,000 and *τ*_*i*_ = 0.1 in all branches for autosomal data, and *N*_e_ = 750 and *τ*_*i*_ = 0.133 for X-linked data. We also reduced the recombination rate for the X chromosome by a 2/3 factor, because of the absence of recombination in males. We then analyzed 50 replicated datasets consisting of 5,000 SNPs sampled from a single autosome and 5,000 SNPs sampled from a single X chromosome. To vary the extent of LD, we sampled SNPs from the whole chromosomes, or from the first 50 Mb, 20 Mb, or 10 Mb. To mimic more realistic datasets, we considered a “whole-genome” sampling scheme, where 5,000 autosomal SNPs were sampled from 20 distinct autosomes and 5,000 X-linked SNPs were sampled from a single X chromosome. As a matter of comparison, we also analyzed 50 datasets simulated with msprime, but assuming strictly independent SNPs.

### Data

#### Cattle data

The analyzed cattle dataset consisted of 60 individuals (55 bulls and 5 cows) belonging to the Holstein dairy cattle breed (HOL), 42 individuals (39 bulls and 3 cows) belonging to the Angus beef cattle breed (ANG), and 23 individuals (4 bulls and 19 cows) belonging to the N’Dama breed (NDA). The data were taken from the public database WIDDE [78], based on the high-density Illumina 770K SNP chip https://www.illumina.com/Documents/products/datasheets/datasheet_bovineHD.pdf. We used DetSex [79] to infer the sex of individuals, and to identify the markers located in the pseudo-autosomal regions of the X chromosome. Only those SNPs that unambiguously mapped to the X chromosome (with posterior probability > 0.95) were retained as X-linked markers. Only those markers that were polymorphic in the full sample, and typed in all sampled individuals were retained, resulting in a total of 643,090 autosomal SNPs and 15,009 X-linked SNPs. For both genetic systems, we randomly subsampled 50 pseudo-replicated datasets from the full data, consisting in 5,000 autosomal SNPs and 5,000 X-linked SNPs. We performed the *f*_3_ admixture test on autosomal SNPs [54] and found no evidence of admixture in all three possible tree topologies: *f*_3_(NDA;HOL,ANG) = 0.083 (*z*-score = 473.1), *f*_3_(HOL;ANG,NDA) = 0.027 (*z*-score = 311.0), and *f*_3_(ANG;HOL,NDA) = 0.019 (*z*-score = 214.3).

#### Human HapMap data

We re-analyzed the dataset from Keinan et al. [19, 42] (available from https://reich.hms.harvard.edu/datasets), consisting of 60 European American individuals from Utah, USA (of North European ancestry; CEU), 60 West African individuals from Ibadan, Nigeria (YRI), and 90 East Asian individuals (45 Han Chinese from Beijing, China, and 45 Japanese from Tokyo, Japan; ASN). From the filtered sequences (level 3), we concatenated all available SNPs from the autosomes and X chromosome, respectively, and removed duplicates (multiple annotated sites). We retained 340,909 autosomal and 12,737 X-linked sites that were polymorphic in the full sample, which we randomly subsampled into 50 pseudo-replicated datasets made of 5,000 autosomal SNPs and 5,000 X-linked SNPs. We performed the *f*_3_ admixture test [54] using autosomal data and found no evidence of admixture in all three possible tree topologies: *f*_3_(CEU;ASN,YRI) = 0.014 (*z*-score = 163.4), *f*_3_(ASN;CEU,YRI) = 0.023 (*z*-score = 216.3), and *f*_3_(YRI;CEU,ASN) = 0.042 (*z*-score = 319.5).

#### Human whole-genome sequence data

The analyzed dataset consisted in a subset of individuals that were recently published in Pagani et al. [33], which combined 379 previously unpublished genomes with sequences from the Personal Genomes Project (http://www.personalgenomes.org) and previously published data from Drmanac et al. [61] and Clemente et al. [80] (available from http://evolbio.ut.ee/CGgenomes.html). All samples have been sequenced at >40x coverage, mapped and called by Complete Genomics (Mountain View, California, USA) using CG software versions 1.5; 2.0, 2.2 and 2.4. Despite minor differences between the pipeline versions, the resulting data can be considered as single platform data with negligible platform bias. The raw data were reduced to contain only SNPs of high quality and were further subject to three subsequent filtering steps: (a) Hardy-Weinberg equilibrium filter, (b) biallelic filter and (c) no-call filter (see Pagani et al. [33] for details).

We were interested in inferring ESR in populations that experienced the Out-of-Africa bottleneck but are genetically as distinct as possible. Using the ADMIXTURE plots from Extended Data Figure 1C in Pagani et al. [33], we clustered together 9 North-West Europeans with 29 Estonians (NW-Europe); 8 Dusun, 9 Murut and 8 Igorot (SE-Asia island); 3 Kosipe and 3 Koinanbe (Oceania) and 5 Cachi, 19 Colla, and 4 Wichi (Americas). Among these individuals, we extracted strictly bi-allelic SNPs (sites with missing data were excluded) that were segregating in the full sample and concatenated the remaining 11,566,865 loci from all autosomes. For the X chromosome, we excluded the pseudo-autosomal regions as annotated in GRCh37.p13 and retained 340,475 X-linked markers. For each genetic system, we then randomly subsampled the full data into 50 pseudo-replicated datasets made of 5,000 autosomal SNPs and 5,000 X-linked SNPs. We performed the four population test (*f*_4_) on all autosomal loci, which suggested no admixture for the unrooted tree (NW-Europe,SE-Asia island); (Oceania,Americas) (*f*_4_ = -0.00019, *z*-score = -1.89). Based on the DIC, we found the star-shaped topology (NW-Europe,SE-Asia,Oceania,Americas) to be the most likely.

## Program availability

The software package containing the C source code and a detailed documentation is freely available for download at http://www1.montpellier.inra.fr/CBGP/software/kimtree/. The code of our generation-by-generation coalescent based simulator, together with all input files that were used to generate the simulated datasets, are available from the Zenodo database [74].

## Supporting information

S1 Text Evaluation of the extended KimTree model. (PDF)

S1 FigIllustration of the constraints that tie the branch lengths for autosomal and X-linked data.This figure shows (within the colored area) the joint support of *τ*^(A)^ and *τ*^(X)^ over the range of possible ESR, since 0 < *ξ* < 1. The support satisfies $9\tau_{i}^{\left(X\right)}/16<\tau_{i}^{\left(A\right)}<9\tau_{i}^{\left(X\right)}/8$ and $8\tau_{i}^{\left(A\right)}/9<\tau_{i}^{\left(X\right)}<16\tau_{i}^{\left(A\right)}/9$. The dashed line indicates the special case *ξ* = 0.5.(TIF)

S2 Fig Performance of the model for estimating branch lengths from full data and SNP-only data. We simulated a four-population tree with topology ((1,2),(3,4)) under the inference model, using a slice-sampling algorithm and assuming a Beta(1,1) distribution for the ancestral allele frequencies. We analyzed 50 replicate simulated datasets made of 5,000 autosomal markers, and *n* = 100 haploid individuals sampled in each population. The boxplots in (A–F) summarize the distributions of the 50 posterior means of *τ*_*i*_ for each of the six branches. Inset trees indicate which branch is considered in each panel. The horizontal dashed line indicates the true (simulated) values of *τ*_*i*_ (*τ*_1_ = *τ*_3_ = *τ*_6_ = 0.1 and *τ*_2_ = *τ*_4_ = *τ*_5_ = 0.05). We ran KimTree on the full data (FD) that included fixed sites. The data were then reduced to polymorphic sites, and we ran analyses assuming a beta distribution with fixed parameters for the ancestral allele frequencies (B(1,1)); we ran analyses where the parameters of the beta distribution were inferred from the data (B(a,b)); last we ran analyses using the conditional likelihood model (CND).
(TIF)

S3 Fig Performance of the model for estimating branch lengths from full data and SNP-only data. We simulated a four-population tree with topology ((1,2),(3,4)) under the inference model, with three-times larger branch lengths as compared to S2 Fig. The boxplots in (A–F) summarize the distributions of the 50 posterior means of *τ*_*i*_ for each of the six branches. Inset trees indicate which branch is considered in each panel. The horizontal dashed line indicates the true (simulated) values of *τ*_*i*_ (*τ*_1_ = *τ*_3_ = *τ*_6_ = 0.3 and *τ*_2_ = *τ*_4_ = *τ*_5_ = 0.15). We ran KimTree on the full data (FD) that included fixed sites; we ran analyses assuming a beta distribution with fixed parameters for the ancestral allele frequencies (B(1,1)); we ran analyses where the parameters of the beta distribution were inferred from the data (B(a,b)); last we ran analyses using the conditional likelihood model (CND).
(TIF)

S4 Fig Comparison of the beta-with-spikes model with various implementations of KimTree. We re-analyzed the 50 SNP datasets simulated by Tataru et al. [48] corresponding to their scenario I. In this scenario, a three-population topology ((1,2),3) was considered with *τ*_1_ = 0.1, *τ*_2_ = *τ*_3_ = 0.133 and *τ*_4_ = 0.2. The ancestral allele frequencies were drawn from a Beta(1,1) distribution, and 5,000 SNPs were simulated with *n* = 100 haploid individuals sampled in each population. The boxplots in (A–D) summarize the distributions of the 50 posterior means of *τ*_*i*_ for each of the four branches. Inset trees indicate which branch is considered in each panel. The horizontal dashed line indicates the true (simulated) values of *τ*_*i*_. The results of Tataru et al. [48] with the beta-with-spikes model is provided (BS); we further ran KimTree analyses assuming a beta distribution with fixed parameters for the ancestral allele frequencies (B(1,1)); we ran analyses where the parameters of the beta distribution were inferred from the data (B(a,b)); last we ran analyses using the conditional likelihood model (CND).
(TIF)

S5 Fig Comparison of the beta-with-spikes model with various implementations of KimTree. We re-analyzed the 50 SNP datasets simulated by Tataru et al. [48] corresponding to their scenario II. In this scenario, a three-population topology ((1,2),3) was considered with *τ*_1_ = 0.044, *τ*_2_ = 0.132, *τ*_3_ = 0.6 and *τ*_4_ = 0.028. The ancestral allele frequencies were drawn from a Beta(0.0188,0.0195) distribution, and 5,000 SNPs were simulated with *n* = 100 haploid individuals sampled in each population. The boxplots in (A–D) summarize the distributions of the 50 posterior means of *τ*_*i*_ for each of the four branches. Inset trees indicate which branch is considered in each panel. The horizontal dashed line indicates the true (simulated) values of *τ*_*i*_. The results of Tataru et al. [48] with the beta-with-spikes model is provided (BS); we further ran KimTree analyses assuming a beta distribution with fixed parameters for the ancestral allele frequencies (B(1,1)); we ran analyses where the parameters of the beta distribution were inferred from the data (B(a,b)); last we ran analyses using the conditional likelihood model (CND).
(TIF)

S6 FigModel assessment.We used the DIC to characterize the strength of evidence for alternative tree topologies, and for alternative models. Autosomal data were generated using ms, as in Gautier and Vitalis [32] assuming a three-population tree with topology $T_{1}^{*}=\left(\left(1,2\right),3\right)$, branch lengths *τ*_*i*_ = 0.1, and 100 genes sampled in each population. 50 replicated datasets were simulated, with a total of 25,000 independent and polymorphic SNPs per replicate. Each dataset was analyzed using either the conditional likelihood model (clear, left-hand side of the graph) or the full likelihood model (shaded, right-hand side of the graph). For each model, either the true topology was considered ($T_{1}^{*})$, or the three possible alternative ones: *T*_2_ = (1,(2,3)), *T*_3_ = ((1,3),2) and *S* = (1, 2, 3). For each condition (i.e., for each column), the colored dots represent the distribution of the DIC rank for the 50 replicated datasets. The size of each dot is proportional to the relative frequency of the corresponding rank, out of 50. For each model, the true topology ($T_{1}^{*}$) correspond to the lowest DIC rank. Furthermore, the conditional likelihood model is favored, relatively to the full likelihood model, whatever topology is considered.(TIF)

S7 FigRobustness to wrong topologies.We reanalyzed the datasets simulated for Fig 2, using either the true topology: $T_{1}^{*}=\left(\left(1,2\right),3\right)$, or the three possible alternative ones: *T*_2_ = (1,(2,3)), *T*_3_ = ((1,3),2) and *S* = (1, 2, 3). As in Fig 2, inset trees indicate which branch was simulated with a biased sex ratio. For each scenario (A, B, C and D), the distributions of the 50 posterior means of *ξ*_*i*_ for each of the three terminal branches are summarized by boxplots. Terminal branches are indeed the only branches that are shared by all possible topologies (branch 1 in red, branch 2 in orange, and branch 3 in green). The horizontal dashed segments indicate the true (simulated) values of *ξ*_*i*_. The pie-charts indicate the fraction of significant support values (*S* < 0.01), against the hypothesis *ξ* = 0.5 (see Eq 4). In A, B and D, the estimated ESR are consistent even when wrong topologies are considered. In C, the estimated ESR for topologies *T*_2_, *T*_3_ and *S* are biased downward, because they integrate over the internal branch where the ESR is biased, yet unaccounted for in the model.(TIF)

S8 FigRobustness to population size change.We simulated two scenarios based on a four-population tree with topology ((1,2),(3,4)), as depicted in the inset tree (top). In all scenarios, the root population was made of 50,000 males and 50,000 females, and the internal branches correspond to populations made of 5,000 males and 5,000 females. The two successive splits occurred 2,000 and 4,000 generations before present time. The mutation rate was fixed at *μ* = 1.5 × 10^−7^. 50 females per population were sampled for each dataset. In (A–C), we simulated an instantaneous 5-fold population growth in branch 1 and an instantaneous 5-fold bottleneck in branch 4, both events having occurred 400 generations before present (as in Fig 2B). In (D–F), we simulated an instantaneous 10-fold population growth in branch 1 and an instantaneous 10-fold bottleneck in branch 4, both events having occurred 400 generations before present. All the other branches corresponded to populations made of 5,000 males and 5,000 females. We analyzed 50 replicate simulated datasets for each scenario, with 5,000 autosomal SNPs and 5,000 X-linked SNPs. The boxplots in (A) and (D) summarize the distributions of the 50 posterior means of $\tau_{i}^{\left(A\right)}$ for each of the six branches. The boxplots in (B) and (E) summarize the distributions of the 50 posterior means of $\tau_{i}^{\left(X\right)}$ for each of the six branches. The boxplots in (C) and (F) summarize the distributions of the 50 posterior means of *ξ*_*i*_ for each of the six branches. In all panels, the horizontal dashed line indicates the true (simulated) values of the parameters. The pie-charts indicate the fraction of significant support values (*S* < 0.01), against the hypothesis *ξ* = 0.5 (see Eq 4).(TIF)

S9 FigRobustness to recent mutations.We simulated two scenarios based on a four-population tree with topology ((1,2),(3,4)), as depicted in the inset tree (top). In all scenarios, all the branches (internal and external) correspond to populations made of 5,000 males and 5,000 females. The two successive splits occurred 2,000 and 4,000 generations before present time. The mutation rate was fixed at *μ* = 1.5 × 10^−7^. 50 females per population were sampled for each dataset. In (A–C), the root population was made of 5,000 males and 5,000 females. In (D–F), the root population was made of 50,000 males and 50,000 females (as in Fig 2A). We analyzed 50 replicate simulated datasets for each scenario, with 5,000 autosomal SNPs and 5,000 X-linked SNPs. The boxplots in (A) and (D) summarize the distributions of the 50 posterior means of $\tau_{i}^{\left(A\right)}$ for each of the six branches. The boxplots in (B) and (E) summarize the distributions of the 50 posterior means of $\tau_{i}^{\left(X\right)}$ for each of the six branches. The boxplots in (C) and (F) summarize the distributions of the 50 posterior means of *ξ*_*i*_ for each of the six branches. In all panels, the horizontal dashed line indicates the true (simulated) values of the parameters. The pie-charts indicate the fraction of significant support values (*S* < 0.01), against the hypothesis *ξ* = 0.5 (see Eq 4).(TIF)

S10 Fig Robustness to linkage disequilibrium. Considering a population history with balanced topology ((1,2),(3,4)), we generated 100 haplotypes of 100 Mb (1 Morgan in our parameterization) for each population and each genetic system, using msprime [77]. Assuming a balanced ESR, we considered *N*_e_ = 1,000 and *τ*_*i*_ = 0.1 in all branches for autosomal data, and *N*_e_ = 750 and *τ*_*i*_ = 0.133 for X-linked data. We also reduced the recombination rate for the X chromosome by a 2/3 factor, because of the absence of recombination in males. We then analyzed 50 replicated datasets consisting of 5,000 SNPs sampled from a single autosome and 5,000 SNPs sampled from a single X chromosome. To vary the extent of LD, we sampled SNPs from the whole chromosomes (100 Mb), or from the first 50 Mb, 20 Mb, or 10 Mb. To mimic more realistic datasets, we considered a “whole-genome” (WG) sampling scheme, where 5,000 autosomal SNPs were sampled from 20 distinct autosomes and 5,000 X-linked SNPs were sampled from a single X chromosome. As a matter of comparison, we also analyzed 50 datasets simulated with msprime, but assuming strictly independent SNPs (“unlnkd”). The boxplots in (A–F) summarize the distributions of the 50 posterior means of *ξ*_*i*_ for each of the six branches. Inset trees indicate which branch is considered in each panel. The horizontal dashed line indicates the true (simulated) values of the parameters. The pie-charts indicate the fraction of significant support values (*S* < 0.01), against the hypothesis *ξ* = 0.5 (see Eq 4).
(TIF)

S11 FigRobustness to SNP ascertainment bias.We simulated a scenario based on a four-population tree with topology ((1,2),(3,4)), as depicted in the inset trees (left). In all scenarios, the root population was made of 50,000 males and 50,000 females, and all the branches (internal and external) correspond to populations made of 5,000 males and 5,000 females (as in Fig 2A). The two successive splits occurred 2,000 and 4,000 generations before present time. The mutation rate was fixed at *μ* = 1.5 × 10^−7^. 50 females per population were sampled for each dataset. Once the data was simulated, we called SNPs using two out of 50 simulated diploids in a panel of populations. Only those sites that were polymorphic in the panel were then considered for the KimTree analysis, using allele counts from the remaining 48 individuals of each sample. We analyzed 50 replicate simulated datasets for each scenario, with 5,000 autosomal SNPs and 5,000 X-linked SNPs. In (A–C), the discovery panel was made of all populations, as depicted with the emphasized branches in the inset tree (top left); in (D–F), the discovery panel was made of populations 1 and 3, as depicted in the inset tree (middle left); in (G–I), the discovery panel was made of populations 1 and 2, as depicted in the inset tree (bottom left). The boxplots in (A), (D) and (G) summarize the distributions of the 50 posterior means of $\tau_{i}^{\left(A\right)}$ for each of the six branches. The boxplots in (B), (E), and (H) summarize the distributions of the 50 posterior means of $\tau_{i}^{\left(X\right)}$ for each of the six branches. The boxplots in (C), (F), and (I) summarize the distributions of the 50 posterior means of *ξ*_*i*_ for each of the six branches. In all panels, the horizontal dashed line indicates the true (simulated) values of the parameters. The pie-charts indicate the fraction of significant support values (*S* < 0.01), against the hypothesis *ξ* = 0.5 (see Eq 4).(TIF)

S12 Fig Robustness to small sample sizes. We simulated replicated datasets following one scenario with balanced sex ratio, based on a four-population tree with topology ((1,2),(3,4)), as depicted in the inset trees. We considered different sampling schemes consisting of 5, 10 or 20 females sampled per population, or 5, 10 or 20 males sampled per population. In all scenarios, the root population was made of 50,000 males and 50,000 females, and the internal branches correspond to populations made of 5,000 males and 5,000 females. The two successive splits occurred 2,000 and 4,000 generations before present time. The mutation rate was fixed at *μ* = 1.5 × 10^−7^. We analyzed 50 replicate simulated datasets for each sampling scheme, with 5,000 autosomal SNPs and 5,000 X-linked SNPs. The boxplots in (A–F) summarize the distributions of the 50 posterior means of *ξ*_*i*_ for each of the six branches. Inset trees indicate which branch is considered in each panel. The horizontal dashed line indicates the true (simulated) values of *ξ*_*i*_. The pie-charts indicate the fraction of significant support values (*S* < 0.01), against the hypothesis *ξ* = 0.5 (see Eq 4).
(TIF)

S13 Fig Application example on human (HapMap) data (pairwise analysis). We re-analyzed the dataset from Keinan et al. [19, 42], with genotypes from European American individuals from Utah, USA (CEU), Asian individuals grouping Han Chinese from Beijing and Japanese from Tokyo (ASN) and Yoruba individuals from Ibadan, Nigeria (YRI) (see the Materials and methods section). Pairwise comparisons between CEU and ASN, CEU and YRI, and ASN and YRI consisted, respectively, in 303,560 (11,054), 335,707 (12,589), and 333,235 (12,399) polymorphic sites for autosomal (X-linked) data. For both genetic systems, we randomly subsampled 50 pseudo-replicated datasets from the full data, each made of 5,000 autosomal SNPs and 5,000 X-linked SNPs. The boxplots in (A–C) summarize the distributions of the posterior means of the ESR for each population in all pairwise comparisons, for the 50 pseudo-replicated datasets. The dotted line indicates the expectation for a balanced ESR (*ξ*_*i*_ = 0.5). The pie-charts indicate the fraction of significant support values (*S* < 0.01) against the hypothesis *ξ* = 0.5 (see Eq 4). (D) The boxplots summarize the distributions of the posterior means of *Q* ≡ *τ*^(A)^/*τ*^(X)^, for each pairwise comparison, for the 50 pseudo-replicated datasets. The dashed line indicates the expectation for a balanced ESR (*Q* = 0.75), and the colored plain segments indicate the estimates obtained by Keinan et al. [19]. We interpret this result, as in Keinan et al. [19], as the consequence of male-biased ESR after the out-of-Africa event and before the split of Europeans and Asians.
(TIF)

S14 FigRobustness to small sample sizes in the whole-genome human sequence data.We simulated a star-tree topology (1,2,3,4) mimicking the subset of the whole-genome sequence data from Pagani et al. [33], with populations from NW-Europe (NWE), SE-Asia (SEA), Oceania (OCE) and Americas (AME). We simulated autosomal branch lengths equal to their estimated values from the real data (Europe: ${\bar{\tau }}_{\text{NWE}}=0.076$; Asia: ${\bar{\tau }}_{\text{SEA}}=0.093$; Oceania: ${\bar{\tau }}_{\text{OCE}}=0.252$ and the Americas: ${\bar{\tau }}_{\text{AME}}=0.127$), assuming balanced ESR and using the true male and female sample sizes. The root population was made of 50,000 males and 50,000 females. The tree in (A) is represented with branch lengths averaged over the 50 posterior means of $\tau_{i}^{\left(A\right)}$ from 50 replicate datasets. The boxplots in (B) summarize the corresponding distributions of the 50 posterior means of *ξ*_*i*_ for each of the four branches. The horizontal dashed line indicates the true (simulated) values of the parameters. The pie-charts indicate the fraction of significant support values (*S* < 0.01), against the hypothesis *ξ* = 0.5 (see Eq 4).(TIF)
