# Supplementary material for: Inferring sex-specific demographic history from SNP data
Source: PLoS Genet. 2018 Jan 31;14(1):e1007191. doi: 10.1371/journal.pgen.1007191 (PMC5809101; doi:10.1371/journal.pgen.1007191)
Supplement: S1 Text — (PDF) [file pgen.1007191.s001.pdf]

S1 Text: Evaluation of the extended KimTree model

Using simulations under the inference model, we evaluated the performance of the three different models, (i) fixed beta distribution (original model) (ii) estimated beta distribution and (iii) estimated beta distribution with conditional likelihood. To evaluate the impact of disregarding sites that are monomorphic across all samples, we simulated a full dataset, consisting of polymorphic sites and sites that were ancestrally polymorphic but got fixed in all samples. Ancestrally monomorphic sites carry no information about the branch lengths of the tree and were not considered. Note that ancestrally polymorphic sites that appear as monomorphic in all samples and ancestrally monomorphic sites can only be distinguished in simulated data sets. In real data sets, however, the true amount of monomorphic sites carrying information about branch lengths is not known.

With the full data set, we could estimate all branch lengths accurately independently of which KimTree model was used (see S2 Fig and S3 Fig). However, when the data was reduced to polymorphic sites, only external branch lengths could be estimated accurately by all models. Internal branch lengths were underestimated by the original KimTree model as observed previously. This deviation could be decreased by estimating the parameters of the beta distribution in the root population. The model using a likelihood conditional on polymorphic sites further improved parameter inference and led to unbiased parameter estimates under the inference model. The similar behavior of the three models for the full dataset but the different performance

for polymorphic data clearly shows that disregarding monomorphic sites has a notable effect on the parameter estimation and thus may explain previous difficulties of the original model.

We further compared the performance of KimTree to the beta with spikes model of Tataru et al. [1], using the simulated datasets from their study. Consistent with their results, we found the beta with spikes model performing better than the original KimTree model on internal and short branches. However, the improved KimTree model using the conditional likelihood performed best in all comparisons (see S4 Fig and S5 Fig).

References

[1] Tataru P, Bataillon T, Hobolth A. Inference under a Wright-Fisher model using an accurate beta approximation. Genetics. 2015;201(3):1133–1141.
