# Supplementary figures and images for: Inferring sex-specific demographic history from SNP data
Source: PLoS Genet. 2018 Jan 31;14(1):e1007191. doi: 10.1371/journal.pgen.1007191 (PMC5809101; doi:10.1371/journal.pgen.1007191)

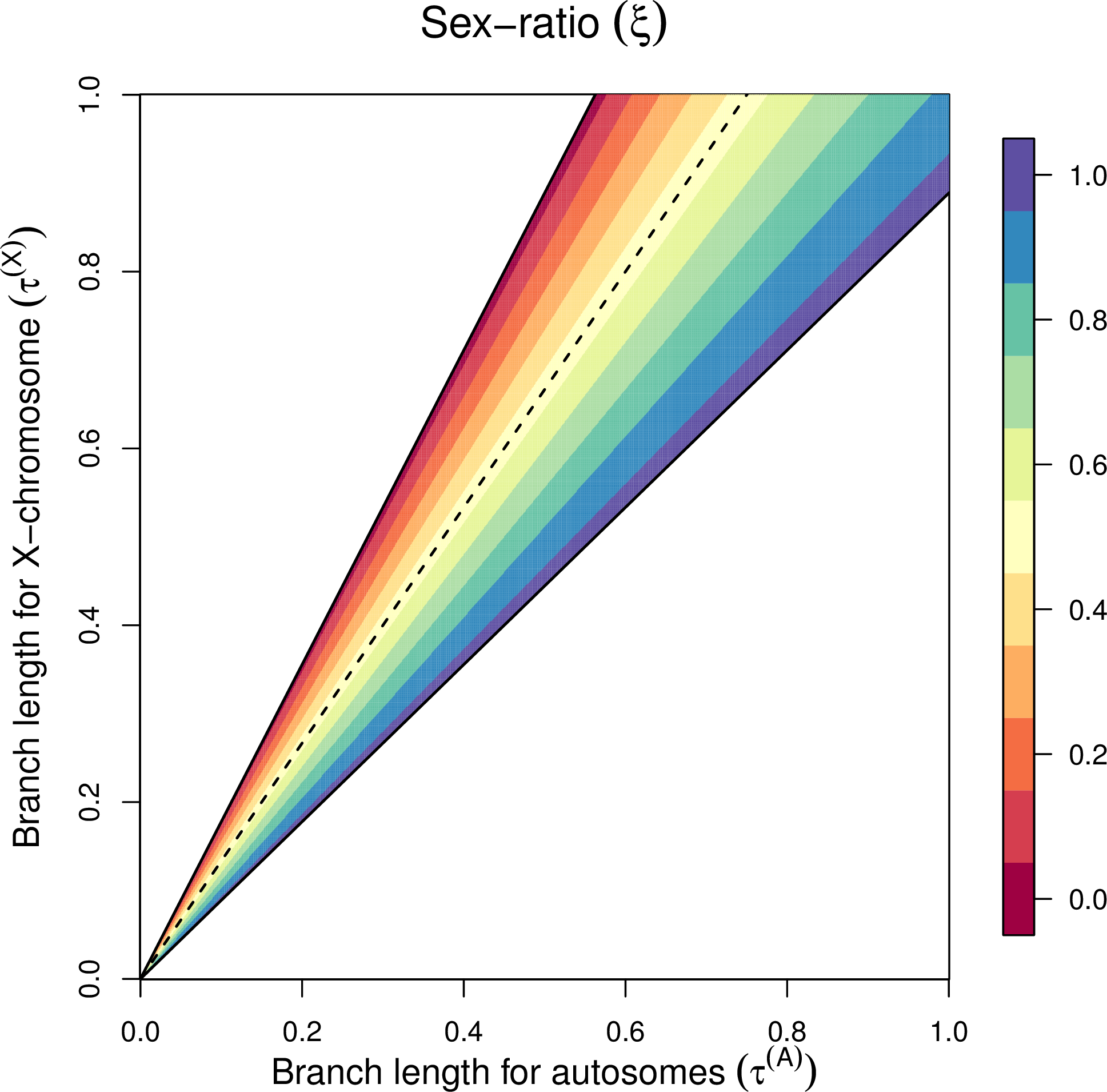

Supplement: S1 Fig — This figure shows (within the colored area) the joint support of τ(A) and τ(X) over the range of possible ESR, since 0 < ξ < 1. The support satisfies 9τi(X)/16<τi(A)<9τi(X)/8 and 8τi(A)/9<τi(X)<16τi(A)/9. The dashed line indicates the special case ξ = 0.5. (TIF) [file pgen.1007191.s002.tif]

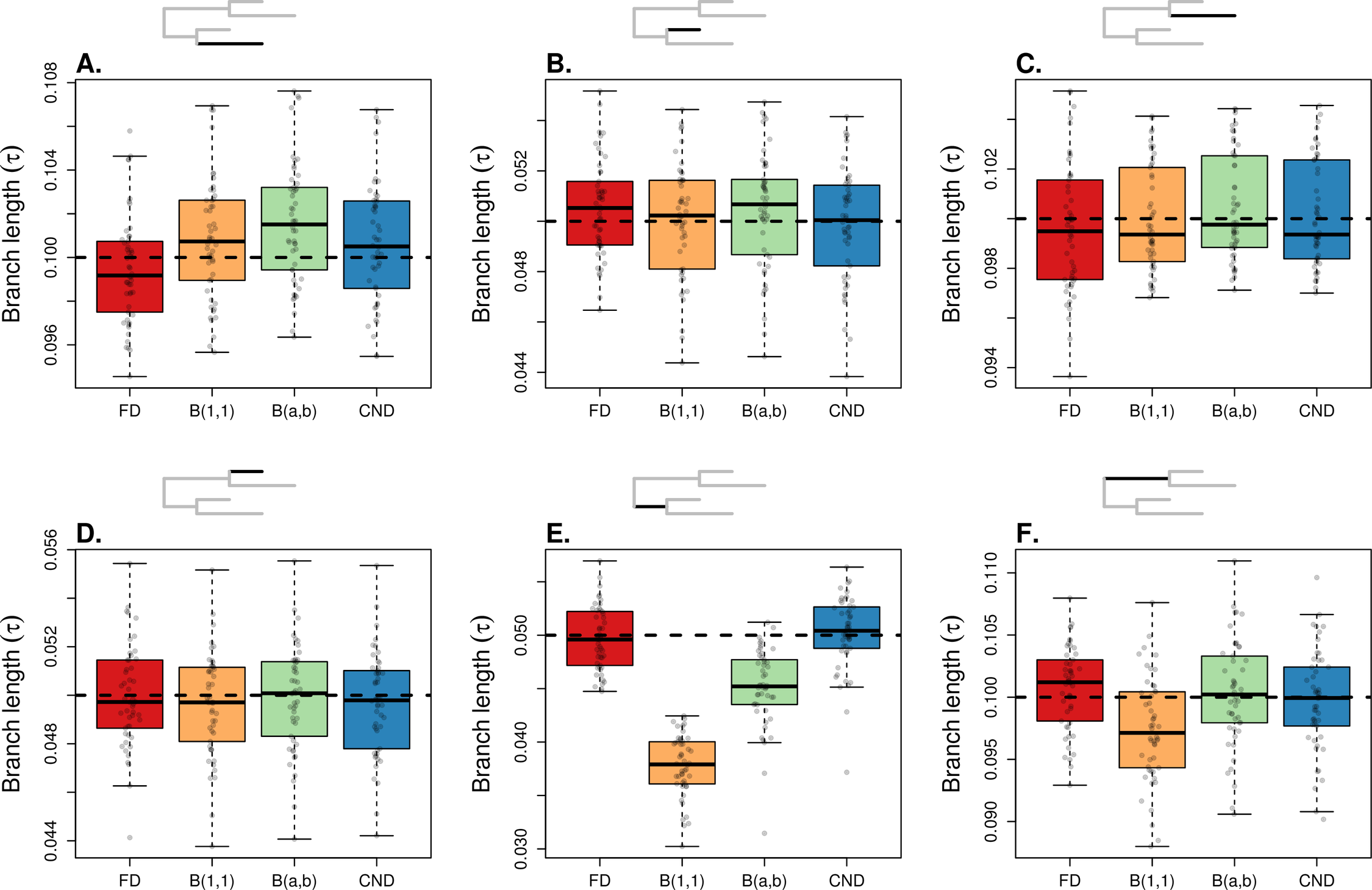

Supplement: S2 Fig — We simulated a four-population tree with topology ((1,2),(3,4)) under the inference model, using a slice-sampling algorithm and assuming a Beta(1,1) distribution for the ancestral allele frequencies. We analyzed 50 replicate simulated datasets made of 5,000 autosomal markers, and n = 100 haploid individuals sampled in each population. The boxplots in (A–F) summarize the distributions of the 50 posterior means of τi for each of the six branches. Inset trees indicate which branch is considered in each panel. The horizontal dashed line indicates the true (simulated) values of τi (τ1 = τ3 = τ6 = 0.1 and τ2 = τ4 = τ5 = 0.05). We ran KimTree on the full data (FD) that included fixed sites. The data were then reduced to polymorphic sites, and we ran analyses assuming a beta distribution with fixed parameters for the ancestral allele frequencies (B(1,1)); we ran analyses where the parameters of the beta distribution were inferred from the data (B(a,b)); last we ran analyses using the conditional likelihood model (CND). (TIF) [file pgen.1007191.s003.tif]

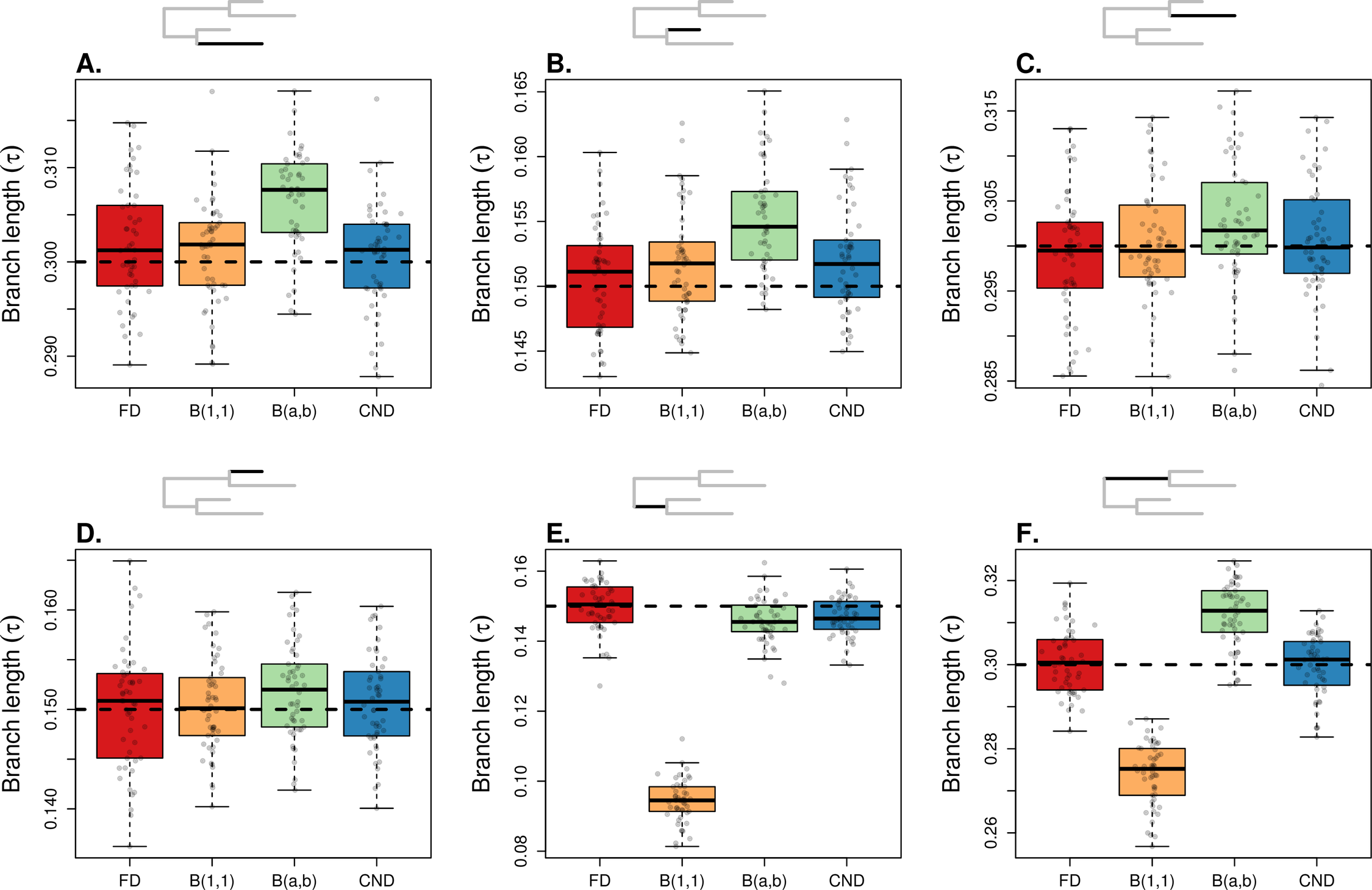

Supplement: S3 Fig — We simulated a four-population tree with topology ((1,2),(3,4)) under the inference model, with three-times larger branch lengths as compared to S2 Fig. The boxplots in (A–F) summarize the distributions of the 50 posterior means of τi for each of the six branches. Inset trees indicate which branch is considered in each panel. The horizontal dashed line indicates the true (simulated) values of τi (τ1 = τ3 = τ6 = 0.3 and τ2 = τ4 = τ5 = 0.15). We ran KimTree on the full data (FD) that included fixed sites; we ran analyses assuming a beta distribution with fixed parameters for the ancestral allele frequencies (B(1,1)); we ran analyses where the parameters of the beta distribution were inferred from the data (B(a,b)); last we ran analyses using the conditional likelihood model (CND). (TIF) [file pgen.1007191.s004.tif]

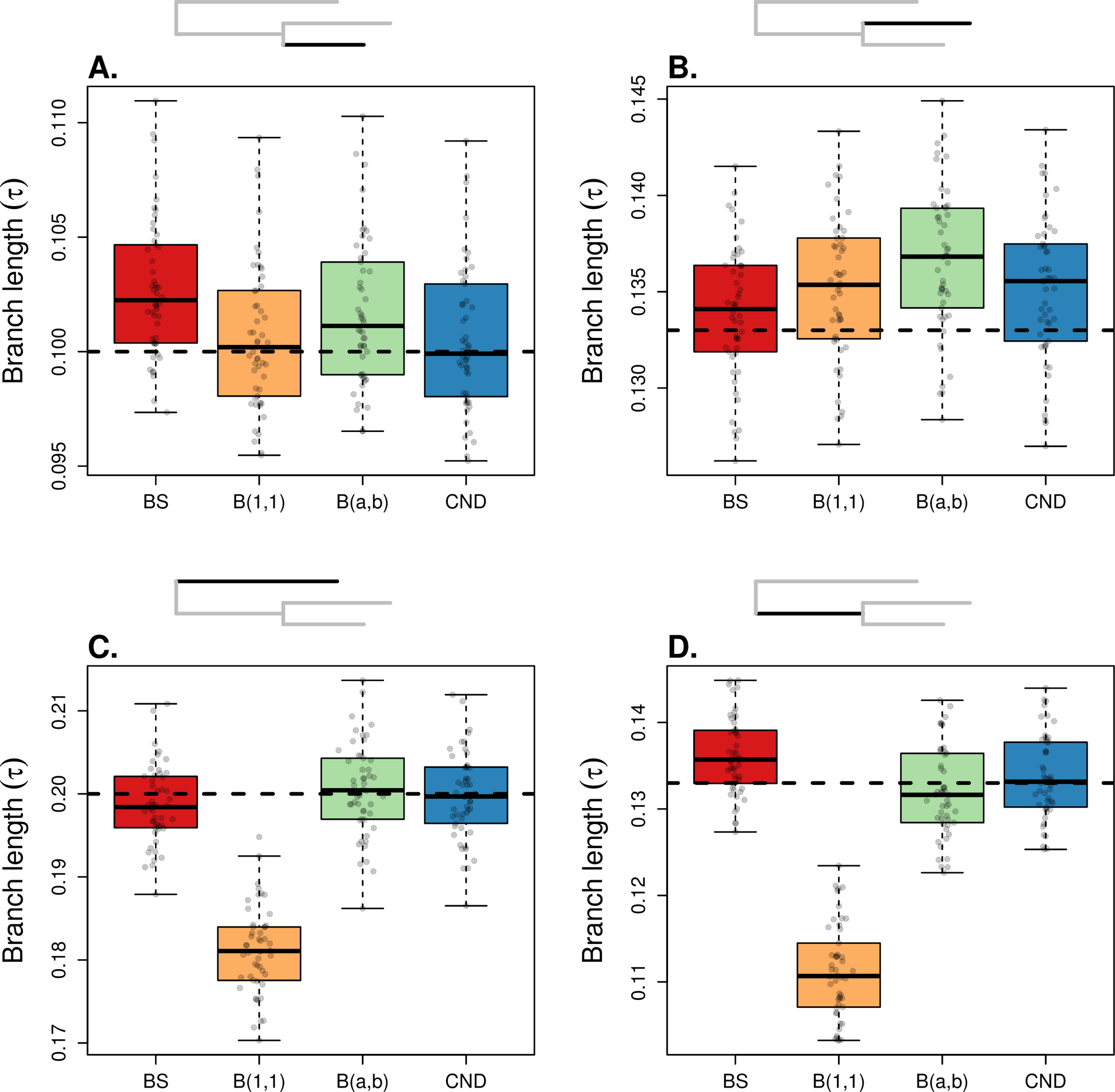

Supplement: S4 Fig — We re-analyzed the 50 SNP datasets simulated by Tataru et al. [48] corresponding to their scenario I. In this scenario, a three-population topology ((1,2),3) was considered with τ1 = 0.1, τ2 = τ3 = 0.133 and τ4 = 0.2. The ancestral allele frequencies were drawn from a Beta(1,1) distribution, and 5,000 SNPs were simulated with n = 100 haploid individuals sampled in each population. The boxplots in (A–D) summarize the distributions of the 50 posterior means of τi for each of the four branches. Inset trees indicate which branch is considered in each panel. The horizontal dashed line indicates the true (simulated) values of τi. The results of Tataru et al. [48] with the beta-with-spikes model is provided (BS); we further ran KimTree analyses assuming a beta distribution with fixed parameters for the ancestral allele frequencies (B(1,1)); we ran analyses where the parameters of the beta distribution were inferred from the data (B(a,b)); last we ran analyses using the conditional likelihood model (CND). (TIF) [file pgen.1007191.s005.tif]

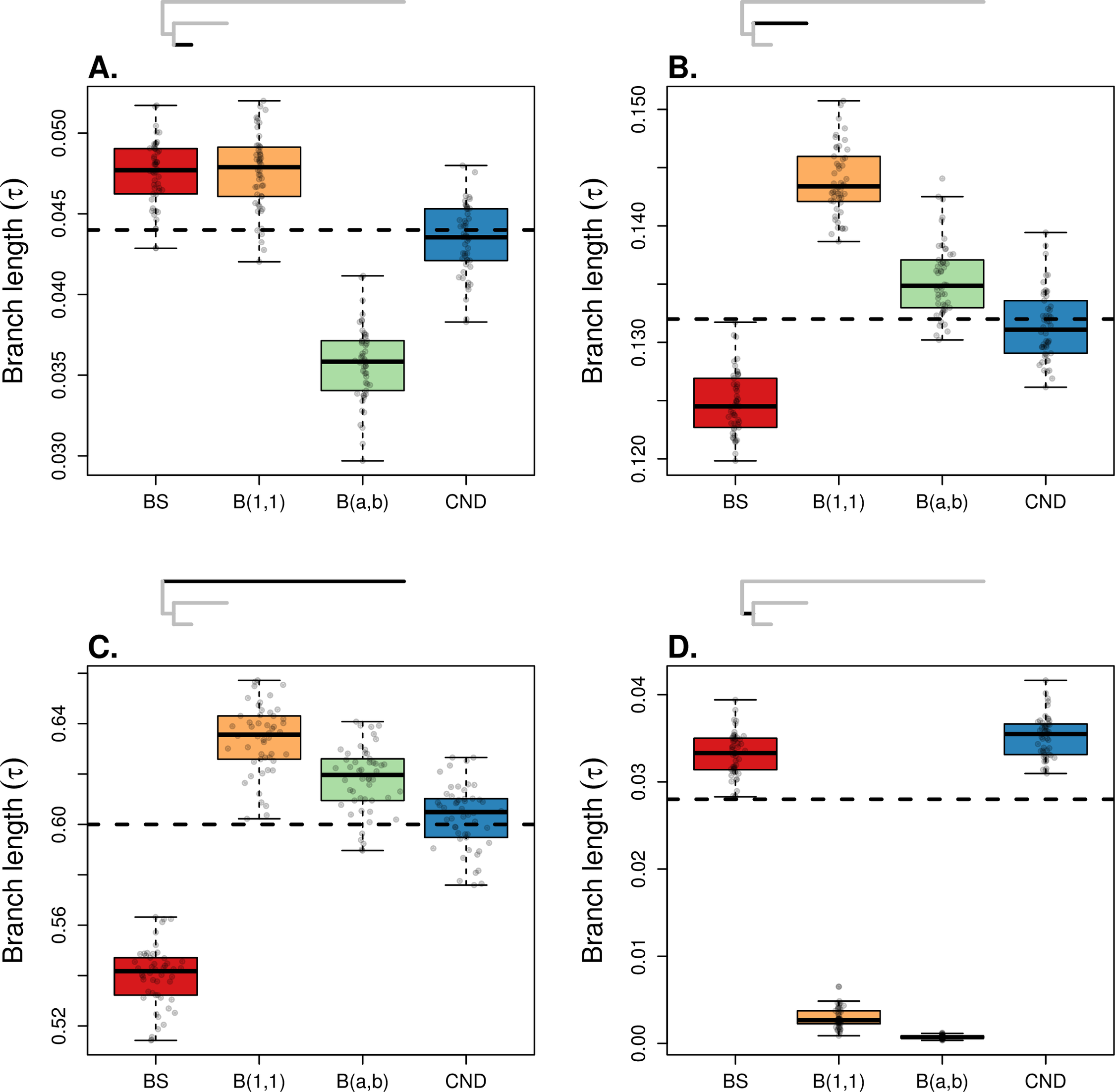

Supplement: S5 Fig — We re-analyzed the 50 SNP datasets simulated by Tataru et al. [48] corresponding to their scenario II. In this scenario, a three-population topology ((1,2),3) was considered with τ1 = 0.044, τ2 = 0.132, τ3 = 0.6 and τ4 = 0.028. The ancestral allele frequencies were drawn from a Beta(0.0188,0.0195) distribution, and 5,000 SNPs were simulated with n = 100 haploid individuals sampled in each population. The boxplots in (A–D) summarize the distributions of the 50 posterior means of τi for each of the four branches. Inset trees indicate which branch is considered in each panel. The horizontal dashed line indicates the true (simulated) values of τi. The results of Tataru et al. [48] with the beta-with-spikes model is provided (BS); we further ran KimTree analyses assuming a beta distribution with fixed parameters for the ancestral allele frequencies (B(1,1)); we ran analyses where the parameters of the beta distribution were inferred from the data (B(a,b)); last we ran analyses using the conditional likelihood model (CND). (TIF) [file pgen.1007191.s006.tif]

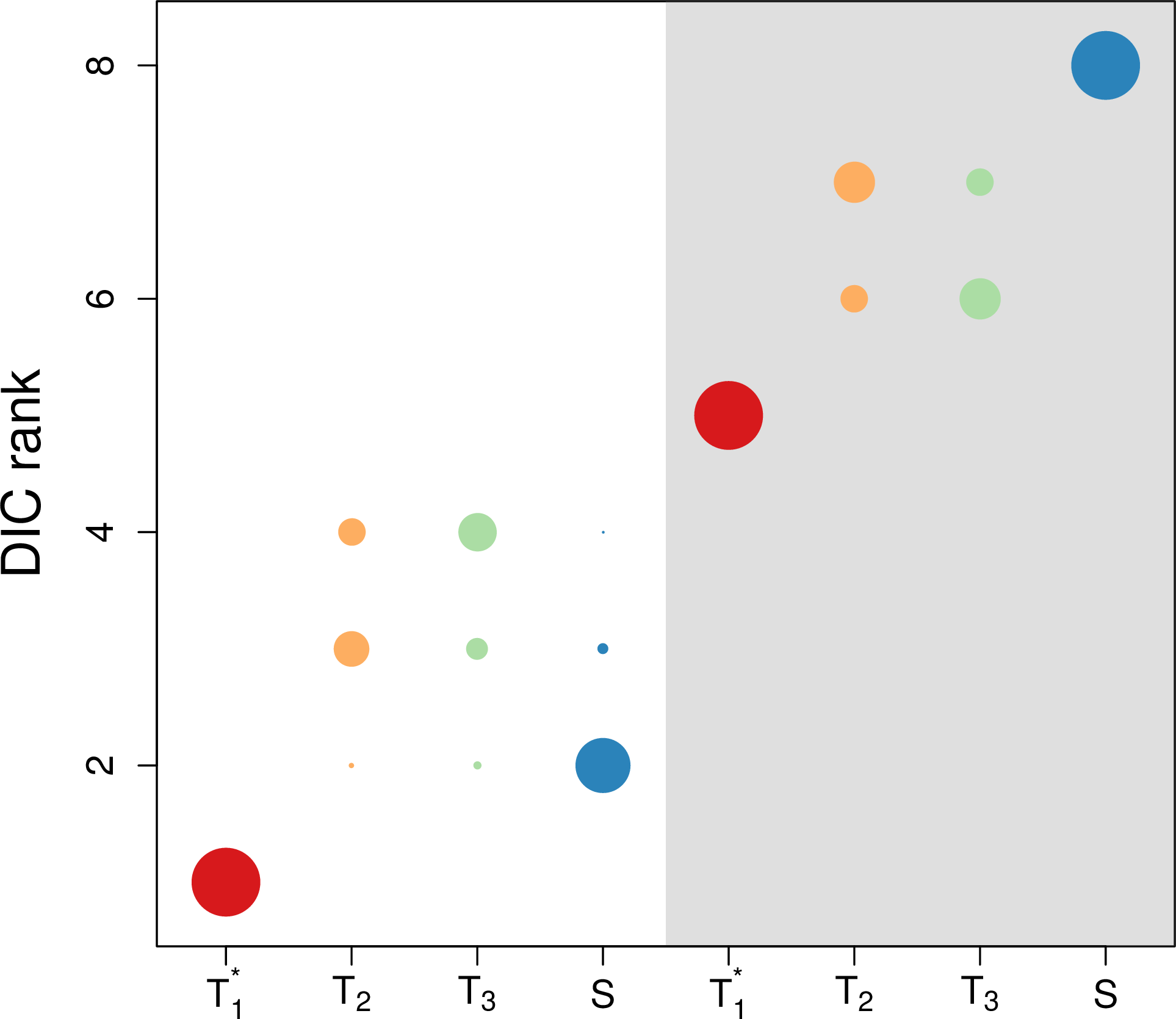

Supplement: S6 Fig — We used the DIC to characterize the strength of evidence for alternative tree topologies, and for alternative models. Autosomal data were generated using ms, as in Gautier and Vitalis [32] assuming a three-population tree with topology T1*=((1,2),3), branch lengths τi = 0.1, and 100 genes sampled in each population. 50 replicated datasets were simulated, with a total of 25,000 independent and polymorphic SNPs per replicate. Each dataset was analyzed using either the conditional likelihood model (clear, left-hand side of the graph) or the full likelihood model (shaded, right-hand side of the graph). For each model, either the true topology was considered (T1*), or the three possible alternative ones: T2 = (1,(2,3)), T3 = ((1,3),2) and S = (1, 2, 3). For each condition (i.e., for each column), the colored dots represent the distribution of the DIC rank for the 50 replicated datasets. The size of each dot is proportional to the relative frequency of the corresponding rank, out of 50. For each model, the true topology (T1*) correspond to the lowest DIC rank. Furthermore, the conditional likelihood model is favored, relatively to the full likelihood model, whatever topology is considered. (TIF) [file pgen.1007191.s007.tif]

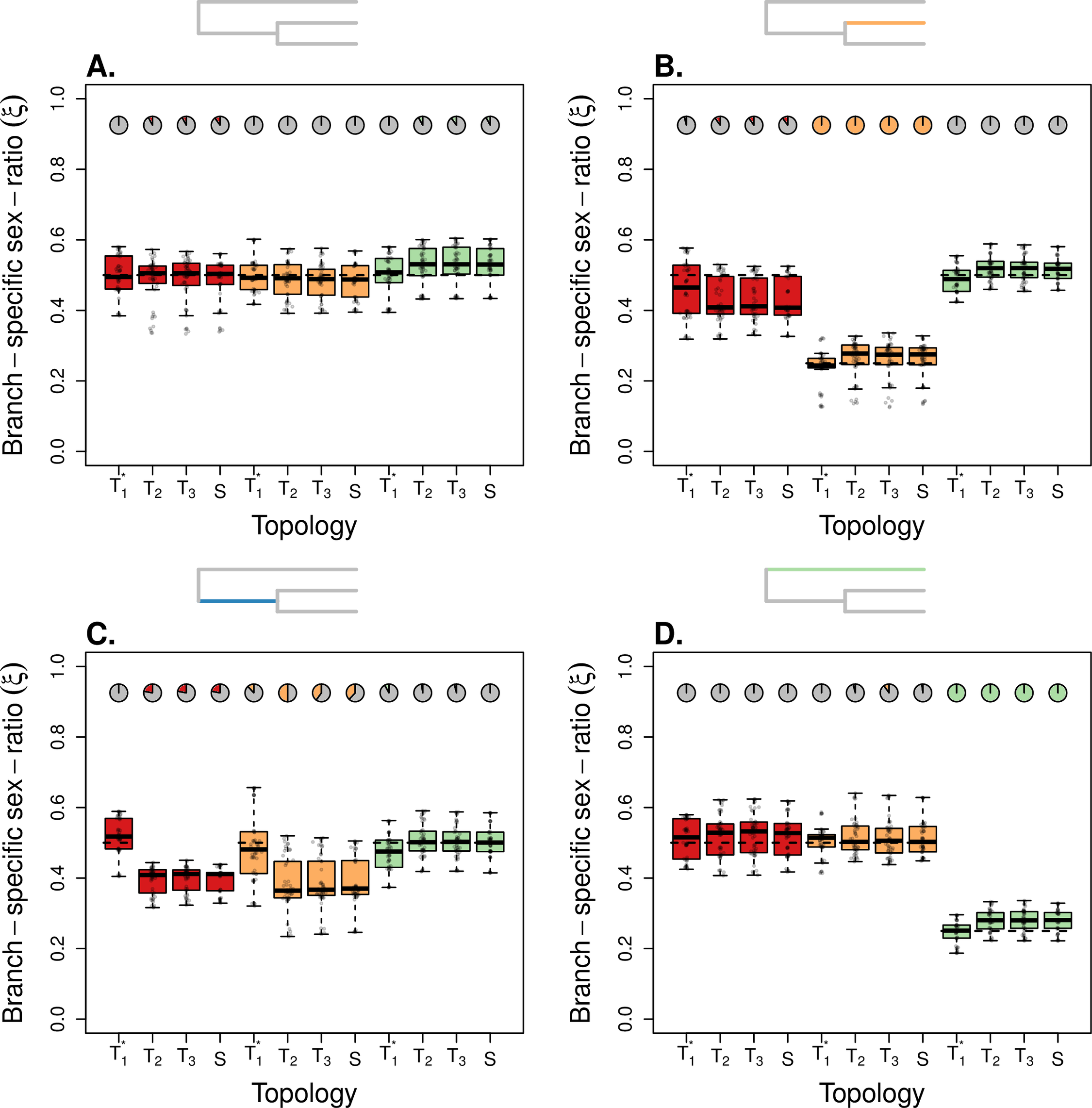

Supplement: S7 Fig — We reanalyzed the datasets simulated for Fig 2, using either the true topology: T1*=((1,2),3), or the three possible alternative ones: T2 = (1,(2,3)), T3 = ((1,3),2) and S = (1, 2, 3). As in Fig 2, inset trees indicate which branch was simulated with a biased sex ratio. For each scenario (A, B, C and D), the distributions of the 50 posterior means of ξi for each of the three terminal branches are summarized by boxplots. Terminal branches are indeed the only branches that are shared by all possible topologies (branch 1 in red, branch 2 in orange, and branch 3 in green). The horizontal dashed segments indicate the true (simulated) values of ξi. The pie-charts indicate the fraction of significant support values (S < 0.01), against the hypothesis ξ = 0.5 (see Eq 4). In A, B and D, the estimated ESR are consistent even when wrong topologies are considered. In C, the estimated ESR for topologies T2, T3 and S are biased downward, because they integrate over the internal branch where the ESR is biased, yet unaccounted for in the model. (TIF) [file pgen.1007191.s008.tif]

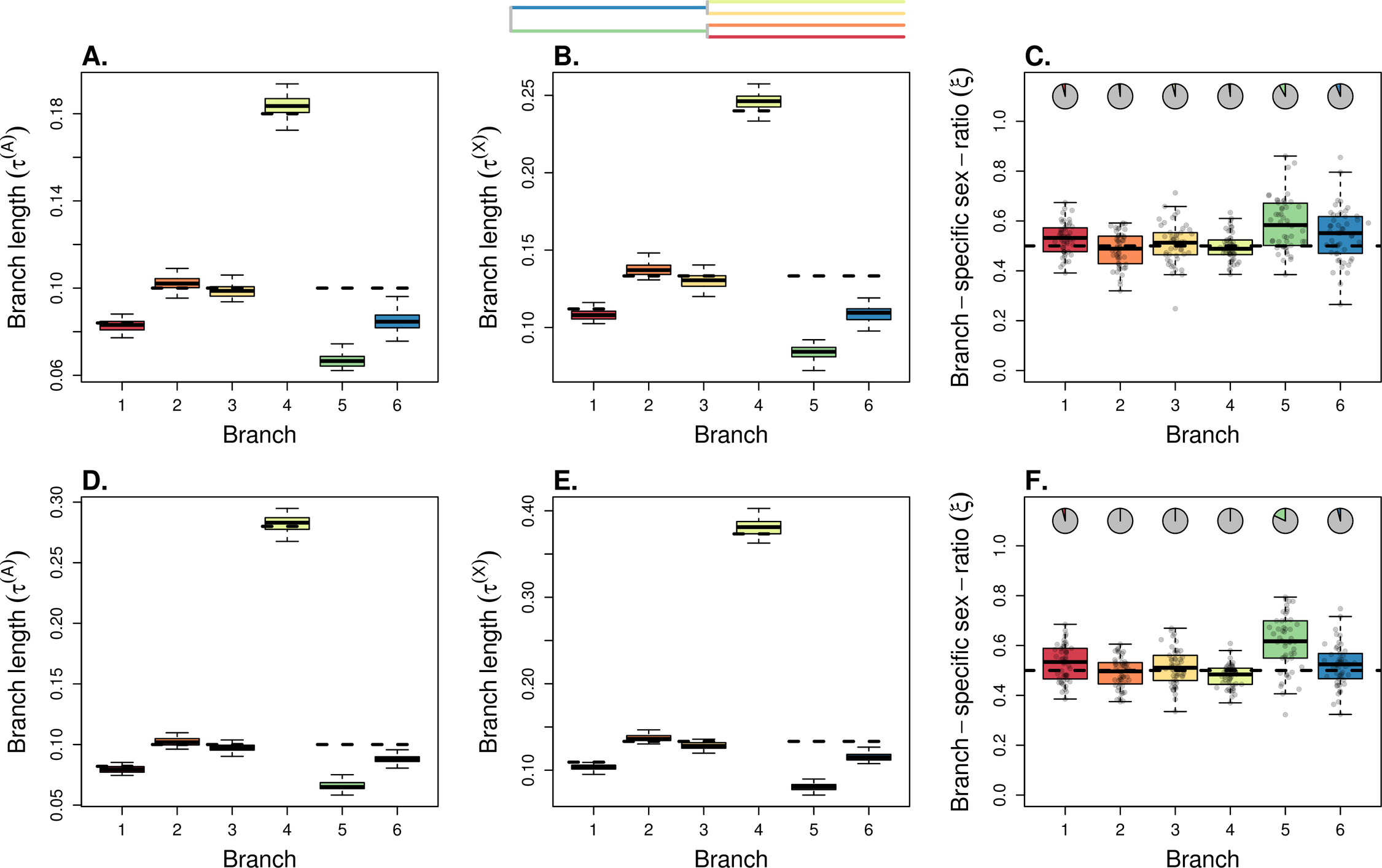

Supplement: S8 Fig — We simulated two scenarios based on a four-population tree with topology ((1,2),(3,4)), as depicted in the inset tree (top). In all scenarios, the root population was made of 50,000 males and 50,000 females, and the internal branches correspond to populations made of 5,000 males and 5,000 females. The two successive splits occurred 2,000 and 4,000 generations before present time. The mutation rate was fixed at μ = 1.5 × 10^−7^. 50 females per population were sampled for each dataset. In (A–C), we simulated an instantaneous 5-fold population growth in branch 1 and an instantaneous 5-fold bottleneck in branch 4, both events having occurred 400 generations before present (as in Fig 2B). In (D–F), we simulated an instantaneous 10-fold population growth in branch 1 and an instantaneous 10-fold bottleneck in branch 4, both events having occurred 400 generations before present. All the other branches corresponded to populations made of 5,000 males and 5,000 females. We analyzed 50 replicate simulated datasets for each scenario, with 5,000 autosomal SNPs and 5,000 X-linked SNPs. The boxplots in (A) and (D) summarize the distributions of the 50 posterior means of τi(A) for each of the six branches. The boxplots in (B) and (E) summarize the distributions of the 50 posterior means of τi(X) for each of the six branches. The boxplots in (C) and (F) summarize the distributions of the 50 posterior means of ξi for each of the six branches. In all panels, the horizontal dashed line indicates the true (simulated) values of the parameters. The pie-charts indicate the fraction of significant support values (S < 0.01), against the hypothesis ξ = 0.5 (see Eq 4). (TIF) [file pgen.1007191.s009.tif]

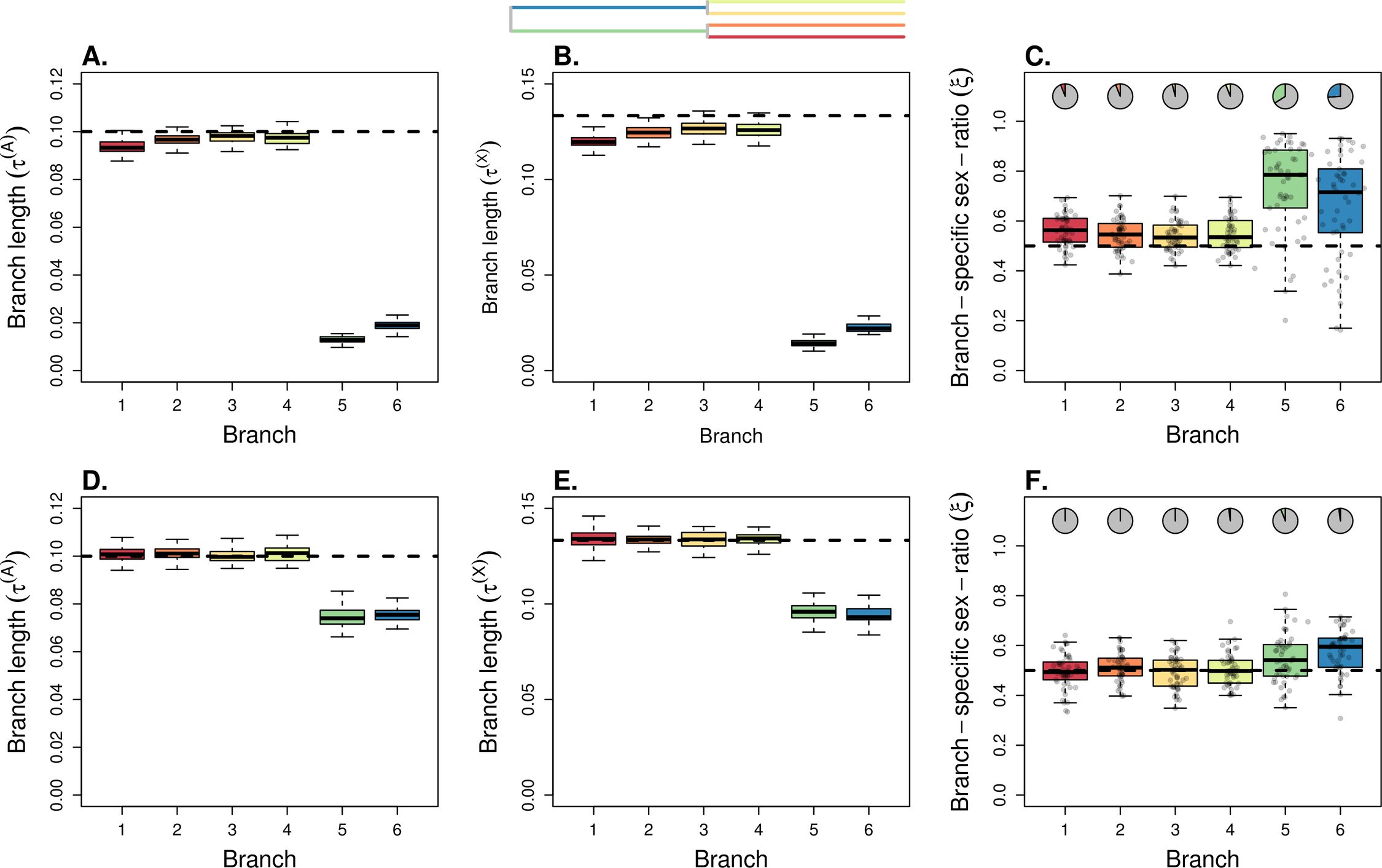

Supplement: S9 Fig — We simulated two scenarios based on a four-population tree with topology ((1,2),(3,4)), as depicted in the inset tree (top). In all scenarios, all the branches (internal and external) correspond to populations made of 5,000 males and 5,000 females. The two successive splits occurred 2,000 and 4,000 generations before present time. The mutation rate was fixed at μ = 1.5 × 10^−7^. 50 females per population were sampled for each dataset. In (A–C), the root population was made of 5,000 males and 5,000 females. In (D–F), the root population was made of 50,000 males and 50,000 females (as in Fig 2A). We analyzed 50 replicate simulated datasets for each scenario, with 5,000 autosomal SNPs and 5,000 X-linked SNPs. The boxplots in (A) and (D) summarize the distributions of the 50 posterior means of τi(A) for each of the six branches. The boxplots in (B) and (E) summarize the distributions of the 50 posterior means of τi(X) for each of the six branches. The boxplots in (C) and (F) summarize the distributions of the 50 posterior means of ξi for each of the six branches. In all panels, the horizontal dashed line indicates the true (simulated) values of the parameters. The pie-charts indicate the fraction of significant support values (S < 0.01), against the hypothesis ξ = 0.5 (see Eq 4). (TIF) [file pgen.1007191.s010.tif]

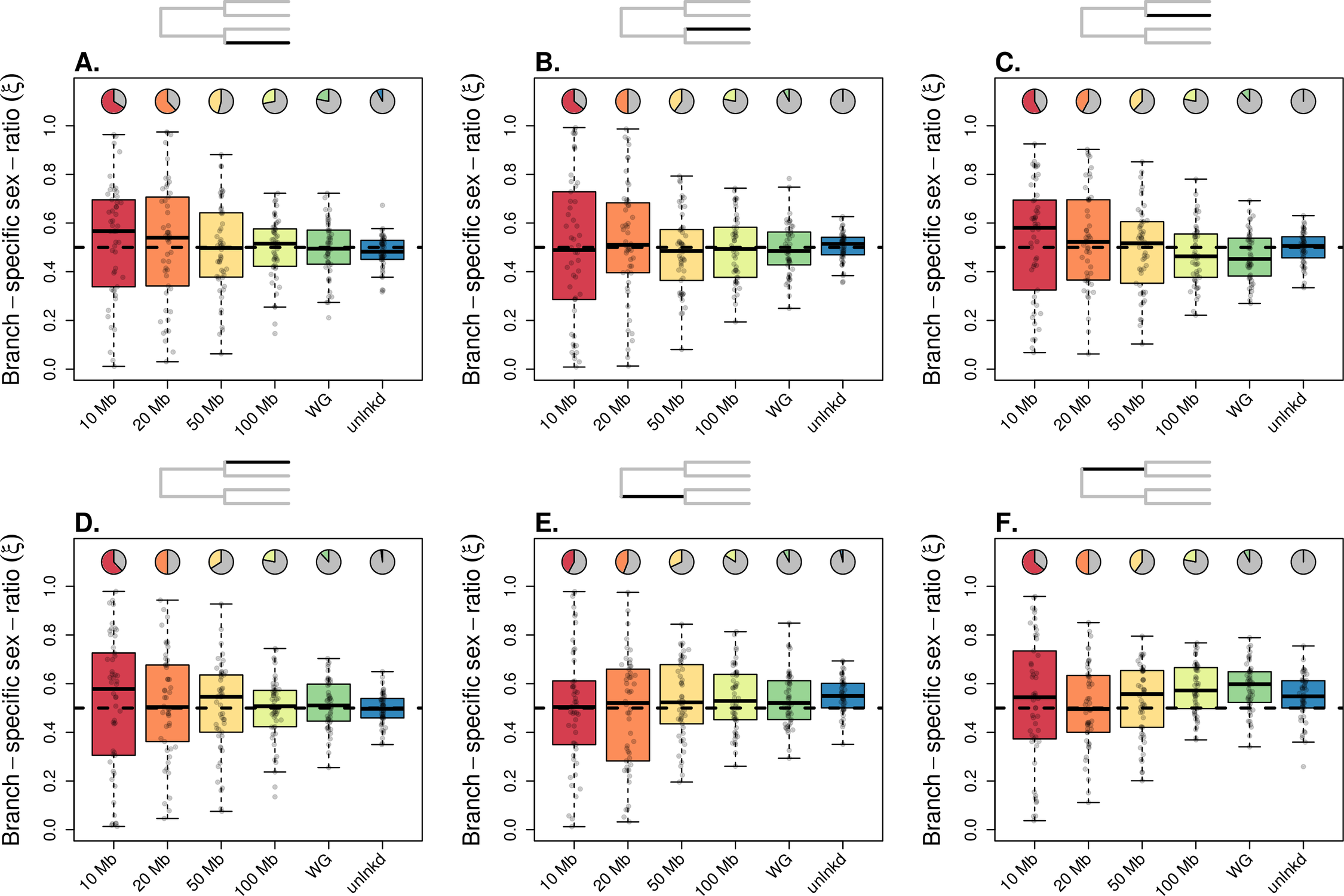

Supplement: S10 Fig — Considering a population history with balanced topology ((1,2),(3,4)), we generated 100 haplotypes of 100 Mb (1 Morgan in our parameterization) for each population and each genetic system, using msprime [77]. Assuming a balanced ESR, we considered Ne = 1,000 and τi = 0.1 in all branches for autosomal data, and Ne = 750 and τi = 0.133 for X-linked data. We also reduced the recombination rate for the X chromosome by a 2/3 factor, because of the absence of recombination in males. We then analyzed 50 replicated datasets consisting of 5,000 SNPs sampled from a single autosome and 5,000 SNPs sampled from a single X chromosome. To vary the extent of LD, we sampled SNPs from the whole chromosomes (100 Mb), or from the first 50 Mb, 20 Mb, or 10 Mb. To mimic more realistic datasets, we considered a “whole-genome” (WG) sampling scheme, where 5,000 autosomal SNPs were sampled from 20 distinct autosomes and 5,000 X-linked SNPs were sampled from a single X chromosome. As a matter of comparison, we also analyzed 50 datasets simulated with msprime, but assuming strictly independent SNPs (“unlnkd”). The boxplots in (A–F) summarize the distributions of the 50 posterior means of ξi for each of the six branches. Inset trees indicate which branch is considered in each panel. The horizontal dashed line indicates the true (simulated) values of the parameters. The pie-charts indicate the fraction of significant support values (S < 0.01), against the hypothesis ξ = 0.5 (see Eq 4). (TIF) [file pgen.1007191.s011.tif]

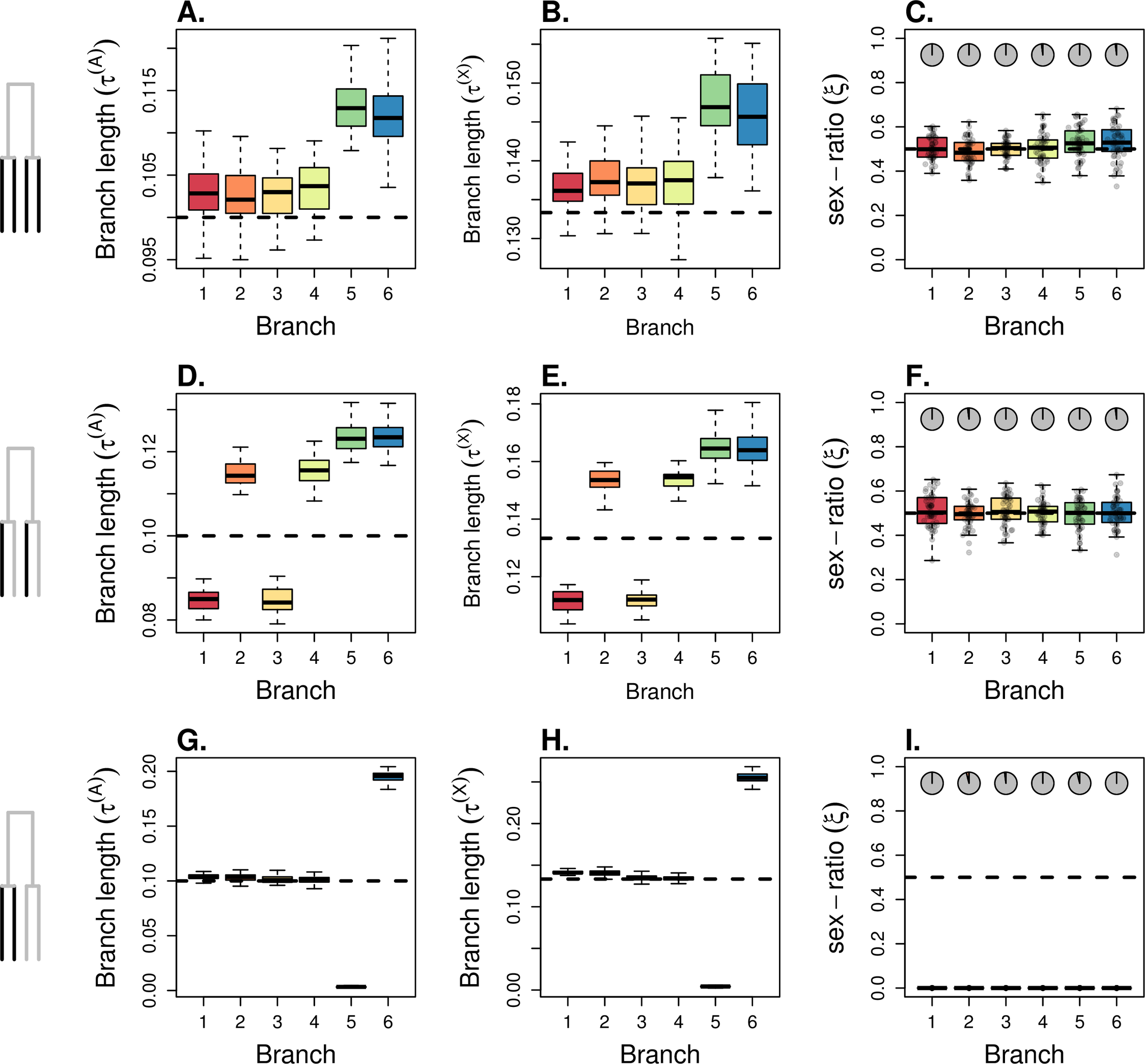

Supplement: S11 Fig — We simulated a scenario based on a four-population tree with topology ((1,2),(3,4)), as depicted in the inset trees (left). In all scenarios, the root population was made of 50,000 males and 50,000 females, and all the branches (internal and external) correspond to populations made of 5,000 males and 5,000 females (as in Fig 2A). The two successive splits occurred 2,000 and 4,000 generations before present time. The mutation rate was fixed at μ = 1.5 × 10^−7^. 50 females per population were sampled for each dataset. Once the data was simulated, we called SNPs using two out of 50 simulated diploids in a panel of populations. Only those sites that were polymorphic in the panel were then considered for the KimTree analysis, using allele counts from the remaining 48 individuals of each sample. We analyzed 50 replicate simulated datasets for each scenario, with 5,000 autosomal SNPs and 5,000 X-linked SNPs. In (A–C), the discovery panel was made of all populations, as depicted with the emphasized branches in the inset tree (top left); in (D–F), the discovery panel was made of populations 1 and 3, as depicted in the inset tree (middle left); in (G–I), the discovery panel was made of populations 1 and 2, as depicted in the inset tree (bottom left). The boxplots in (A), (D) and (G) summarize the distributions of the 50 posterior means of τi(A) for each of the six branches. The boxplots in (B), (E), and (H) summarize the distributions of the 50 posterior means of τi(X) for each of the six branches. The boxplots in (C), (F), and (I) summarize the distributions of the 50 posterior means of ξi for each of the six branches. In all panels, the horizontal dashed line indicates the true (simulated) values of the parameters. The pie-charts indicate the fraction of significant support values (S < 0.01), against the hypothesis ξ = 0.5 (see Eq 4). (TIF) [file pgen.1007191.s012.tif]

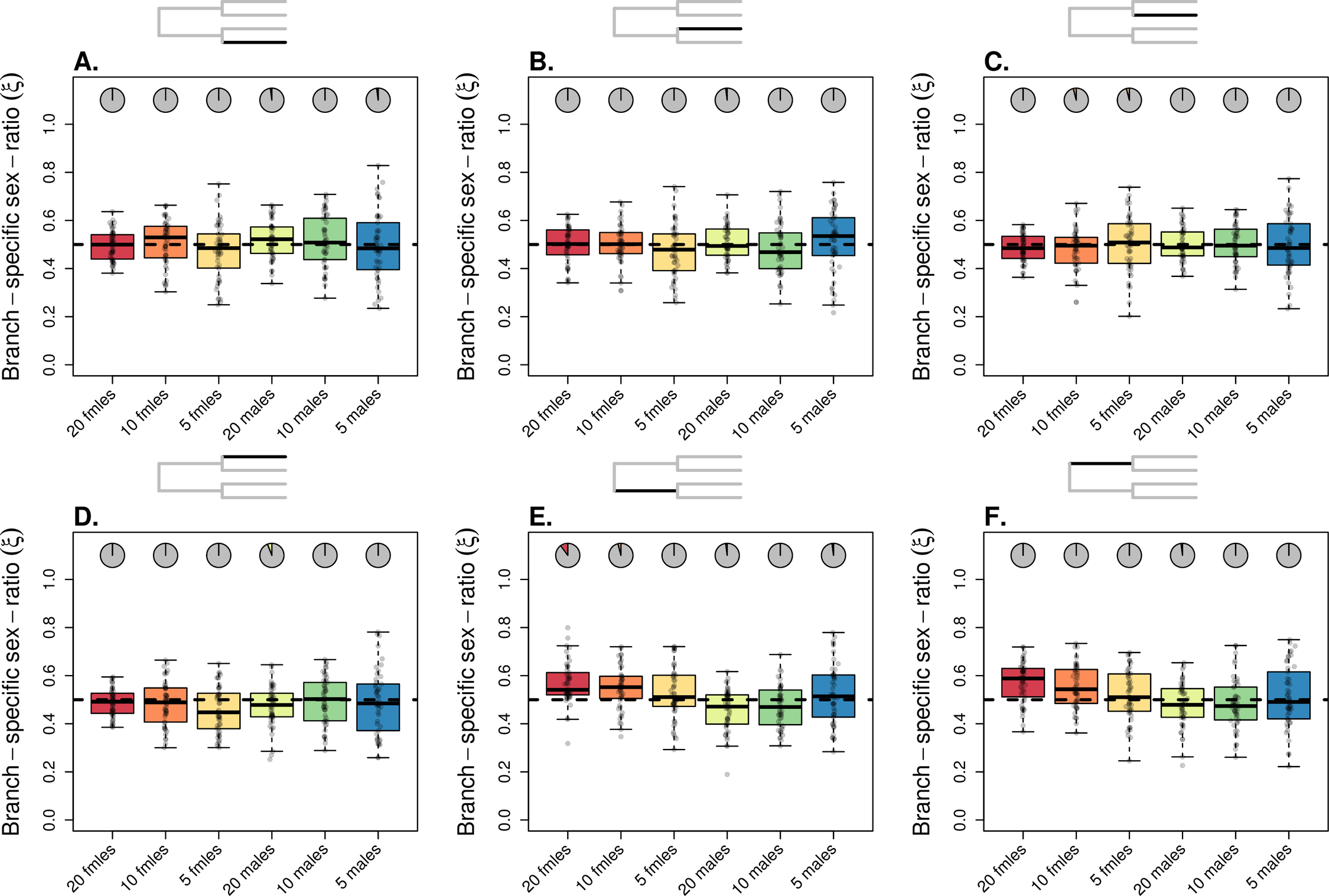

Supplement: S12 Fig — We simulated replicated datasets following one scenario with balanced sex ratio, based on a four-population tree with topology ((1,2),(3,4)), as depicted in the inset trees. We considered different sampling schemes consisting of 5, 10 or 20 females sampled per population, or 5, 10 or 20 males sampled per population. In all scenarios, the root population was made of 50,000 males and 50,000 females, and the internal branches correspond to populations made of 5,000 males and 5,000 females. The two successive splits occurred 2,000 and 4,000 generations before present time. The mutation rate was fixed at μ = 1.5 × 10^−7^. We analyzed 50 replicate simulated datasets for each sampling scheme, with 5,000 autosomal SNPs and 5,000 X-linked SNPs. The boxplots in (A–F) summarize the distributions of the 50 posterior means of ξi for each of the six branches. Inset trees indicate which branch is considered in each panel. The horizontal dashed line indicates the true (simulated) values of ξi. The pie-charts indicate the fraction of significant support values (S < 0.01), against the hypothesis ξ = 0.5 (see Eq 4). (TIF) [file pgen.1007191.s013.tif]

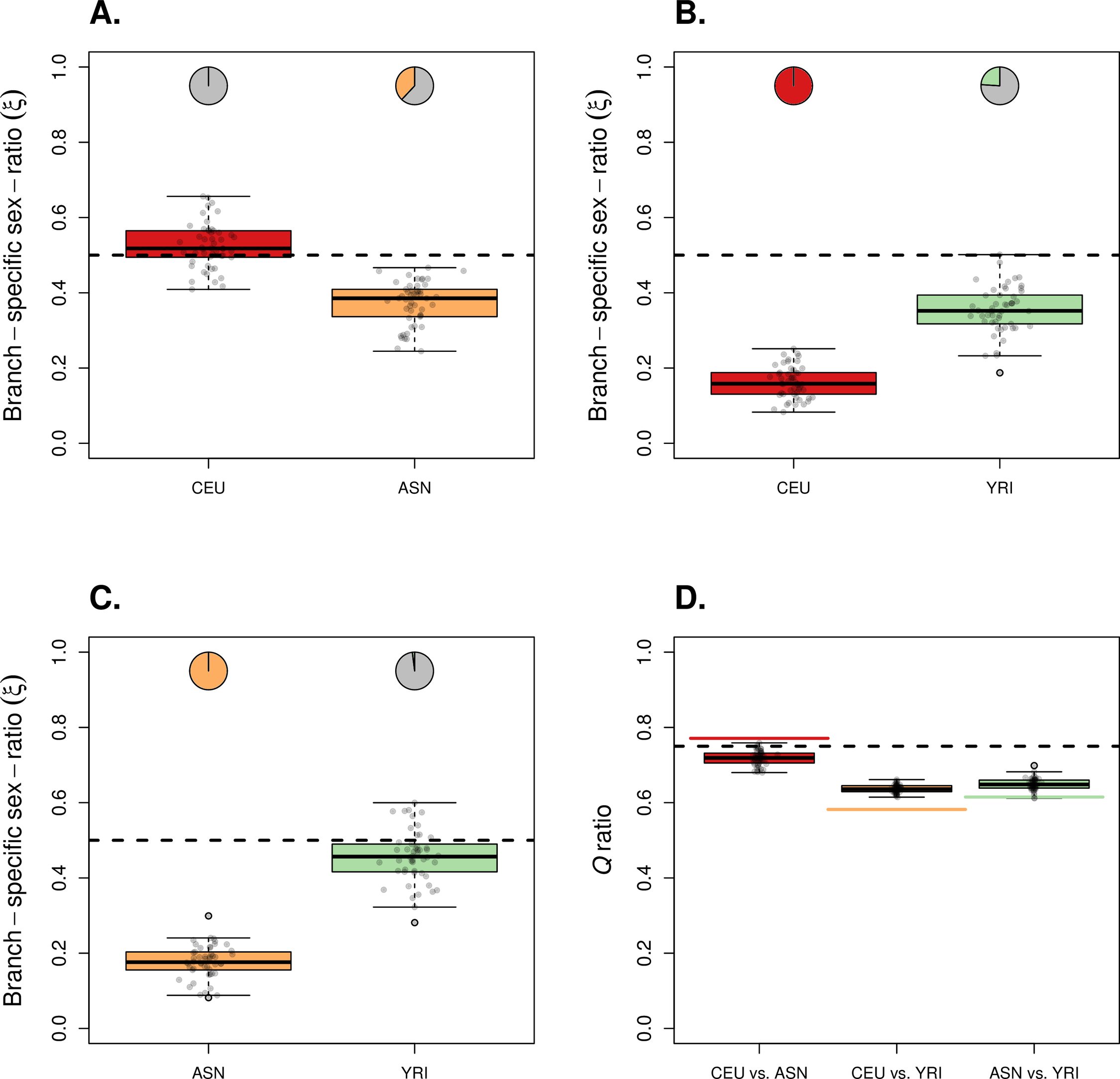

Supplement: S13 Fig — We re-analyzed the dataset from Keinan et al. [19, 42], with genotypes from European American individuals from Utah, USA (CEU), Asian individuals grouping Han Chinese from Beijing and Japanese from Tokyo (ASN) and Yoruba individuals from Ibadan, Nigeria (YRI) (see the Materials and methods section). Pairwise comparisons between CEU and ASN, CEU and YRI, and ASN and YRI consisted, respectively, in 303,560 (11,054), 335,707 (12,589), and 333,235 (12,399) polymorphic sites for autosomal (X-linked) data. For both genetic systems, we randomly subsampled 50 pseudo-replicated datasets from the full data, each made of 5,000 autosomal SNPs and 5,000 X-linked SNPs. The boxplots in (A–C) summarize the distributions of the posterior means of the ESR for each population in all pairwise comparisons, for the 50 pseudo-replicated datasets. The dotted line indicates the expectation for a balanced ESR (ξi = 0.5). The pie-charts indicate the fraction of significant support values (S < 0.01) against the hypothesis ξ = 0.5 (see Eq 4). (D) The boxplots summarize the distributions of the posterior means of Q ≡ τ(A)/τ(X), for each pairwise comparison, for the 50 pseudo-replicated datasets. The dashed line indicates the expectation for a balanced ESR (Q = 0.75), and the colored plain segments indicate the estimates obtained by Keinan et al. [19]. We interpret this result, as in Keinan et al. [19], as the consequence of male-biased ESR after the out-of-Africa event and before the split of Europeans and Asians. (TIF) [file pgen.1007191.s014.tif]

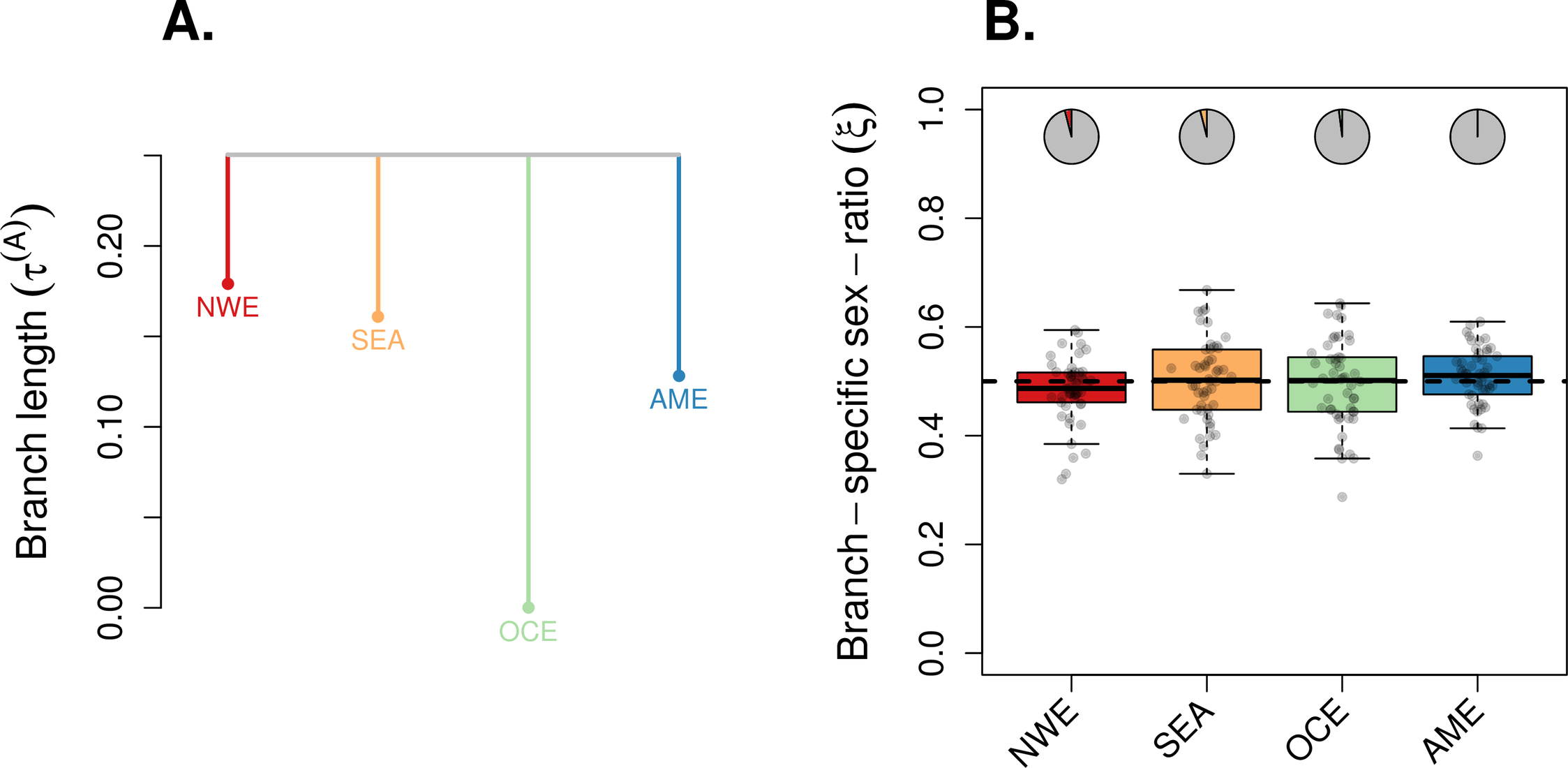

Supplement: S14 Fig — We simulated a star-tree topology (1,2,3,4) mimicking the subset of the whole-genome sequence data from Pagani et al. [33], with populations from NW-Europe (NWE), SE-Asia (SEA), Oceania (OCE) and Americas (AME). We simulated autosomal branch lengths equal to their estimated values from the real data (Europe: τ¯NWE=0.076; Asia: τ¯SEA=0.093; Oceania: τ¯OCE=0.252 and the Americas: τ¯AME=0.127), assuming balanced ESR and using the true male and female sample sizes. The root population was made of 50,000 males and 50,000 females. The tree in (A) is represented with branch lengths averaged over the 50 posterior means of τi(A) from 50 replicate datasets. The boxplots in (B) summarize the corresponding distributions of the 50 posterior means of ξi for each of the four branches. The horizontal dashed line indicates the true (simulated) values of the parameters. The pie-charts indicate the fraction of significant support values (S < 0.01), against the hypothesis ξ = 0.5 (see Eq 4). (TIF) [file pgen.1007191.s015.tif]
